# Synthesis of carbohydrate-scaffolded thymine glycoconjugates to organize multivalency

**DOI:** 10.3762/bjoc.11.75

**Published:** 2015-05-07

**Authors:** Anna K Ciuk, Thisbe K Lindhorst

**Affiliations:** 1 Christiana Albertina University of Kiel, Otto Diels Institute of Organic Chemistry, Otto-Hahn-Platz 3/4, D-24118 Kiel, Germany, Fax: +49 431 8807410

**Keywords:** [2 + 2] photocycloaddition, carbohydrate scaffolds, multivalency, thymine glycoconjugates

## Abstract

Multivalency effects are essential in carbohydrate recognition processes as occurring on the cell surface. Thus many synthetic multivalent glycoconjugates have been developed as important tools for glycobiological research. We are expanding this collection of molecules by the introduction of carbohydrate-scaffolded divalent glycothymine derivatives that can be intramolecularily dimerized by [2 + 2] photocycloaddition. Thus, thymine functions as a control element that allows to restrict the conformational flexibility of the scaffolded sugar ligands and thus to “organize” multivalency. With this work we add a parameter to multivalency studies additional to valency.

## Introduction

Multivalency of molecular interactions is a fundamental principle in carbohydrate recognition. It influences the avidity and specificity of carbohydrate–protein interactions as well as it enables supramolecular changes on the cell surface that are essential for cell–cell communication [1–4]. During the last two decades it has become clear that there is not one mechanism underlying multivalency effects in glycobiology, but that there are a multitude of biological processes involving multivalency in one or the other way. These processes allow to control, regulate and fine-tune the complex life of eukaryotes.

We have recently focused our research dedicated to multivalency effects in carbohydrate recognition on the aspect of conformational control of multivalent assemblies, such as micelles [5] or glycoarrays [6]. This work is based on the idea that changes of ligand orientation as well as changes of their conformational availability are regulating parameters in carbohydrate recognition, in particular on the cell surface. Indeed, we have formerly shown that the molecular dynamics of glycodendrimers influence their recognition by lectins [7]. Recently, we have introduced a photoswitchable glycoazobenzene-covered surface, in which alteration of ligand orientation allowed to switch cell adhesion without changing the recognition quality or the valency of the ligand itself [6]. It is also well-known that galectin-mediated organization of cell surface glycoconjugates influences glycan dynamics and essential biological processes like signaling [8]. Therefore, in order to advance our understanding of carbohydrate-mediated biological response, we are seeking further molecular architectures that allow us organizing the multivalency of sugar ligands.

**Figure 1:**
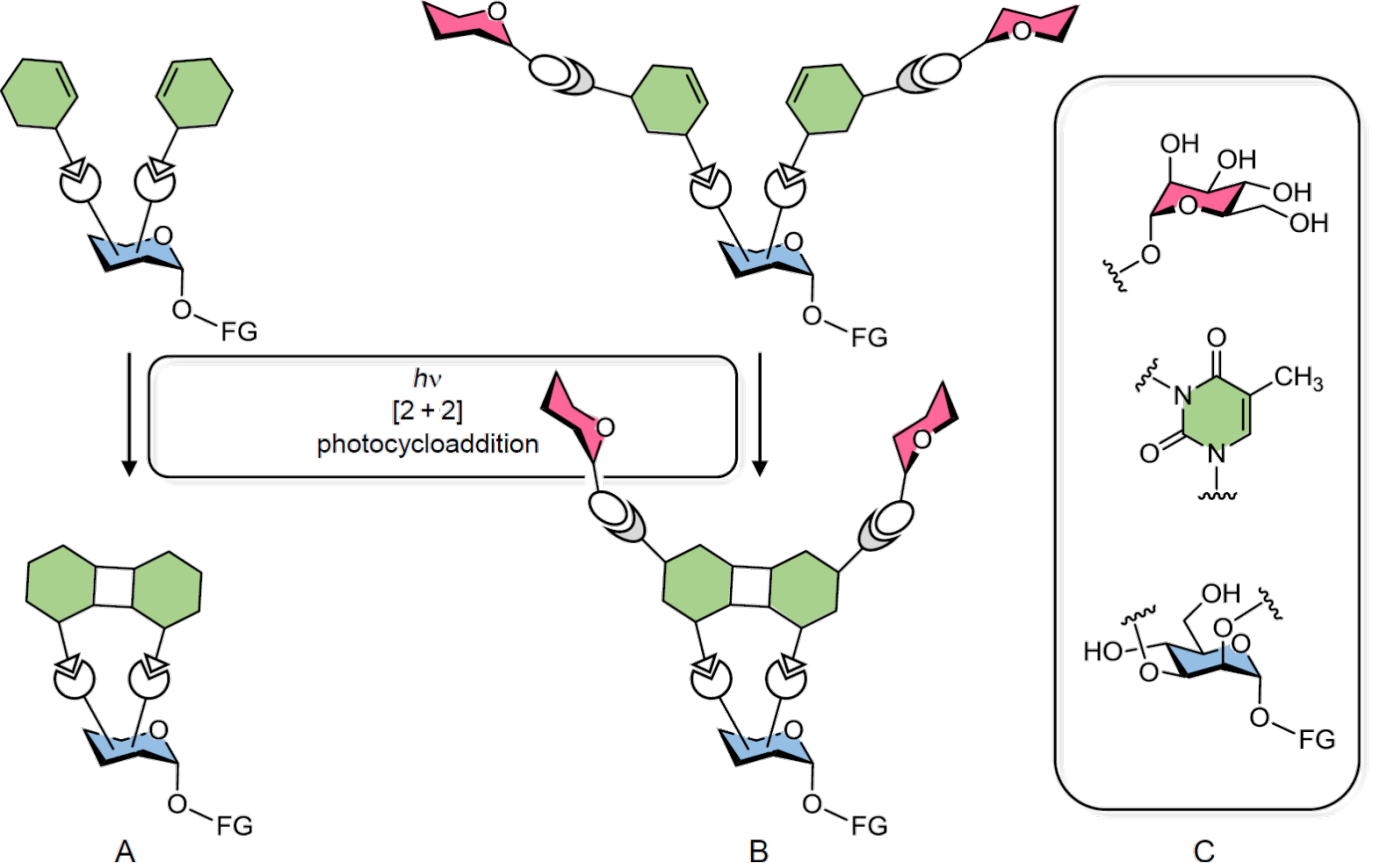
Cartoon of a divalent carbohydrate-scaffolded molecular architecture that allows control of the flexibility of both branches by intramolecular [2 + 2] photocycloaddition (A). To attach the photocontrollable moieties (green) to the carbohydrate scaffold (blue), an appropriate ligation chemistry is required. Additional ligation of sugar ligands (pink) leads to the analogous divalent glycothymine glycoconjugate (B). As building blocks for this molecular architecture a mannoside scaffold (blue), thymine (green), and α-D-mannosyl ligands (pink) were used (C). FG = functional group.

We planned for a divalent system to start with, in which the dynamics of two at first flexible branches can be controlled by an intramolecular [2 + 2] cycloaddition reaction (Figure 1A). In order to control the [2 + 2] cycloaddition process, it was planned to install both branches on a carbohydrate scaffold. This would allow to favour the intramolecular [2 + 2] cycloaddition over an intermolecular reaction and moreover, a multifunctional carbohydrate scaffold facilitates further ligation or immobilization, respectively, of the final molecular construct. After appropriate carbohydrate conjugation the same molecular architecture could be applied for organization of a divalent glycoconjugate (Figure 1B).

We selected thymine as a photocontrollable element as it can undergo [2 + 2] photocycloaddition upon irradiation with light of *h*ν ≥ 290 nm [5,9–10]. Furthermore, it is known that the thymine heterocycle can be easily N^3^-alkylated with, for example, bromoalkyl glycosides. This reaction can be used to install specific sugar moieties for biological recognition [5]. Hence, thymine was employed as the photosensitive control element, a functionalized mannoside was used as scaffold molecule, and α-D-mannosides as specific carbohydrate ligands for the fabrication of the envisaged divalent glycoconjugate (Figure 1C). In the following, we report the synthesis of the divalent glycoconjugates outlined in Figure 1 and their [2 + 2] photocycloaddition.

## Results and Discussion

**Scheme 1:**
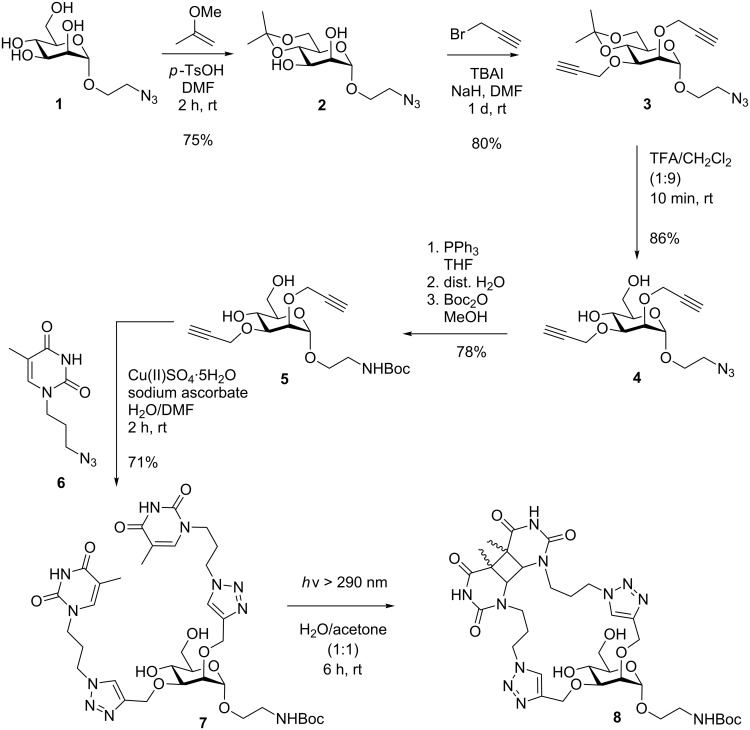
Synthesis of carbohydrate-scaffolded dimeric thymine **7** and intramolecular photocycloaddition. The irradiation product **8** is a complex isomeric mixture (not shown in detail, cf. Supporting Information File 1).

For the conjugation of two thymine and eventually two glycothymine branches on a glycosidic scaffold, the 2- and 3-hydroxy functions of mannose were chosen. These two functional groups can be easily addressed orthogonally to the rest of the molecule. In addition, the vicinal 2- and 3-hydroxy groups are *cis*-oriented and thus the intramolecular [2 + 2] photocycloaddition of attached thymine moieties should be facilitated. As the anomeric functional group of the chosen mannoside scaffold, a Boc-protected amino group was selected which can be derived from an azide function. Thus, the synthesis of the targeted divalent thymine glycoconjugate started with the known 2-azidoethyl α-D-mannoside (**1**, Scheme 1) [11–13]. The kinetically controlled reaction of mannoside **1** with 2-methoxypropene delivers the 4,6-isopropylidene-protected derivative **2** in good yield, leaving the 2- and 3-hydroxy groups free [14–15]. The following Williamson etherification [16] with propargyl bromide yielded the 2,3-di-*O*-propargylmannoside **3** in high yield. Propargylation was selected for this step to allow eventual conjugation with the known azidopropylated thymine derivative **6** [17–20] via copper(I)-catalyzed click reaction [21–22]. Before, the acid-labile isopropylidene protecting group was removed using 10% TFA in dichloromethane leading to mannoside **4**, and then the azide function in **4** was converted in a Staudinger reduction [23] with simultaneous Boc-protection giving rise to mannoside **5** in high overall yield. Boc-protection of the amino group also facilitated the chromatographic purification process in the subsequent steps.

Mannoside **5** is equipped with two propargyl branches projecting from the β-face of the carbohydrate, whereas the Boc-protected aminoethyl aglycone is α-positioned and ready for eventual immobilisation of the final thymine glycoconjugate on a surface or for conjugation to another multivalent compound. For the click reaction of **5** with the thymine derivative **6**, copper sulfate and sodium ascorbate were used. The conjugation product **7** was obtained in 71% yield and showed good water solubility.

**Figure 2:**
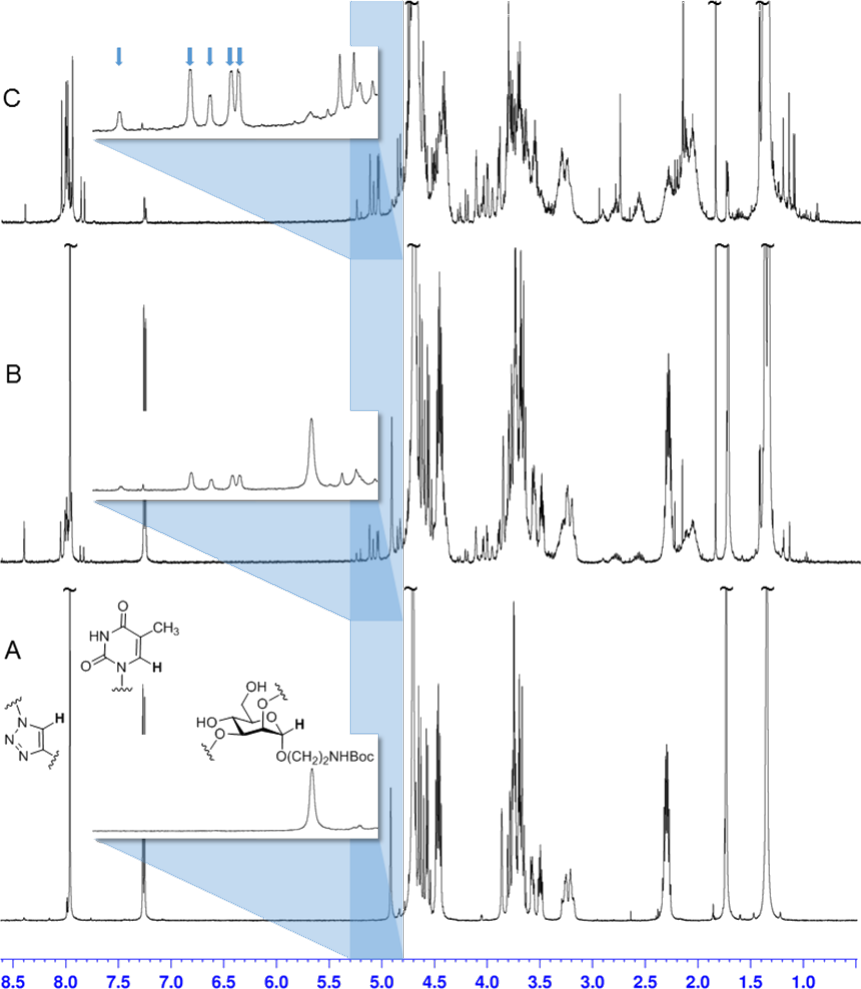
^1^H NMR spectra (all in D_2_O, 500 MHz) of mannoside **7** (A) and of the irradiation product (**8**) after 3 hours irradiation time (B) and 6 hours irradiation time (C). Progress of photocycloaddition is seen in the changes of the signals for the triazole H-11 and the thymine H-6 protons (left at low field) and the anomeric H-1 of the carbohydrate scaffold (≈4.9 ppm). The anomeric H-1 region is detailed in each case: after 6 hours of irradiation (C) five new signals are seen (blue arrows) corresponding to five different isomers of the photocycloaddition product **8**.

Then [2 + 2] photocycloaddition was tested. For irradiation the divalent thymine glycoconjugate **7** was diluted (maximum concentration was 500 μg/1 mL) in a 1:1-mixture of water and acetone in order to favor intramolecular photocycloaddition and avoid the intermolecular reaction. Acetone is required as triplett sensitizer in this process [5]. Irradiation with light of ≥ 290 nm was performed during a period of 3 and 6 hours, respectively. After irradiation, the solvents were removed and the product investigated without any purification or further separation. ^1^H NMR-spectroscopic analysis shows that the signals for the two thymine H-6 protons in **7** at 7.27 and 7.25 ppm decrease during irradiation and finally almost disappear (they are shifted to ≈3.7 ppm in **8**). In addition, the signals for the two triazole H-11 (7.96 ppm) and for the anomeric scaffold H-1 (H-1_core_) at 4.92 ppm get multiplicated. Interestingly, while the H-1 peak of the starting material is clearly diminished, a total of five, slightly downfield-shifted doublets is seen in the [2 + 2] photocycloaddition product **8** (Figure 2C, 5.0–5.3 ppm). These signals correspond to five different isomeric photocycloaddition products.

The [2 + 2] photocycloaddition of thymine derivatives has been extensively discussed in the literature [9–10,24–25] because it is important in DNA damage (as cycloreversion is in DNA repair) [26–27]. Accordingly, the photodimerisation can occur in an *anti* and a *syn* fashion leading to regioisomeric products (cf. Supporting Information File 1). Both regioisomers can consist of four different stereoisomers, two *cis* and two *trans* isomers according to the relative steric orientation of the thymine methyl groups. It can be assumed that the irradiation of **7** in diluted solution favors the *syn* photocycloaddition leading to two isomeric *cis–syn-* and two *trans–syn***-**[2 + 2] photocycloaddition products. In addition, also an *anti* product seems to form as five (not four) H-1 signals are seen in the ^1^H NMR spectrum.

The photocycloaddition of **7** can also be observed by UV–vis spectroscopy. Upon irradiation, the absorption at 270 nm completely disappears (cf. Supporting Information File 1). Mass-spectrometric analysis allows to exclude intermolecular photocycloaddition as no corresponding mass peaks were detected (cf. Supporting Information File 1).

**Scheme 2:**
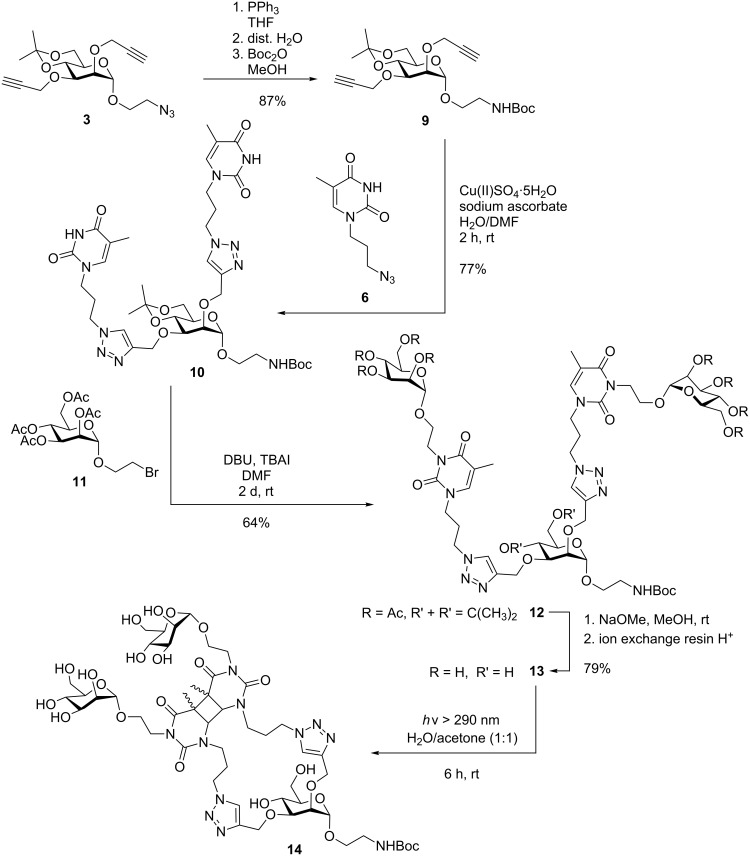
Synthesis of carbohydrate-scaffolded dimeric glycothymine **13** and intramolecular photocycloaddition. The irradiation product **14** is a complex isomeric mixture (not shown in detail, cf. Supporting Information File 1).

In the next step, glycosylated thymine dimers were targeted (cf. Figure 1B). Based on our earlier work [5], we first planned to employ an appropriate bromoalkyl mannoside for *N*-alkylation of the thymine N^3^ [28]. In this step, DBU can be employed as non-nucleophilic base [29] leaving the NHBoc group intact. When sodium hydride is employed instead, NHBoc is deprotonated in addition to thymine and undesired alkylation of NHBoc is occurring. However, also the free hydroxy functions are perturbing the reaction and hence we found no reaction conditions for a clean thymine N^3^ functionalization of **7**. Therefore, an OH-protected analogue of **7** was prepared starting from **3** (Scheme 2). Staudinger reduction of the azide group and Boc-protection in the same pot gave **9** and subsequent click reaction with the thymine derivative **6** the isopropylidene-protected dimeric thymine glycoconjugate **10** in good yield. This could be alkylated employing the bromoethyl mannoside **11** [11] in an optimized procedure employing DBU and TBAI at rt [5] over two days to deliver the protected glycothymine derivative **12** in 64% yield. The acetyl protecting groups were cleaved employing Zemplén’s procedure [30]. During work-up with acidic ionic exchange resin, surprisingly cleavage of the isopropylidene protecting group was observed whereas the Boc-protection remained untouched. Thus, the title glycocluster **13** was obtained in a single deprotection step. The new divalent glycoconjugate **13** shows good water solubility and is thus suited for biological testing.

**Figure 3:**
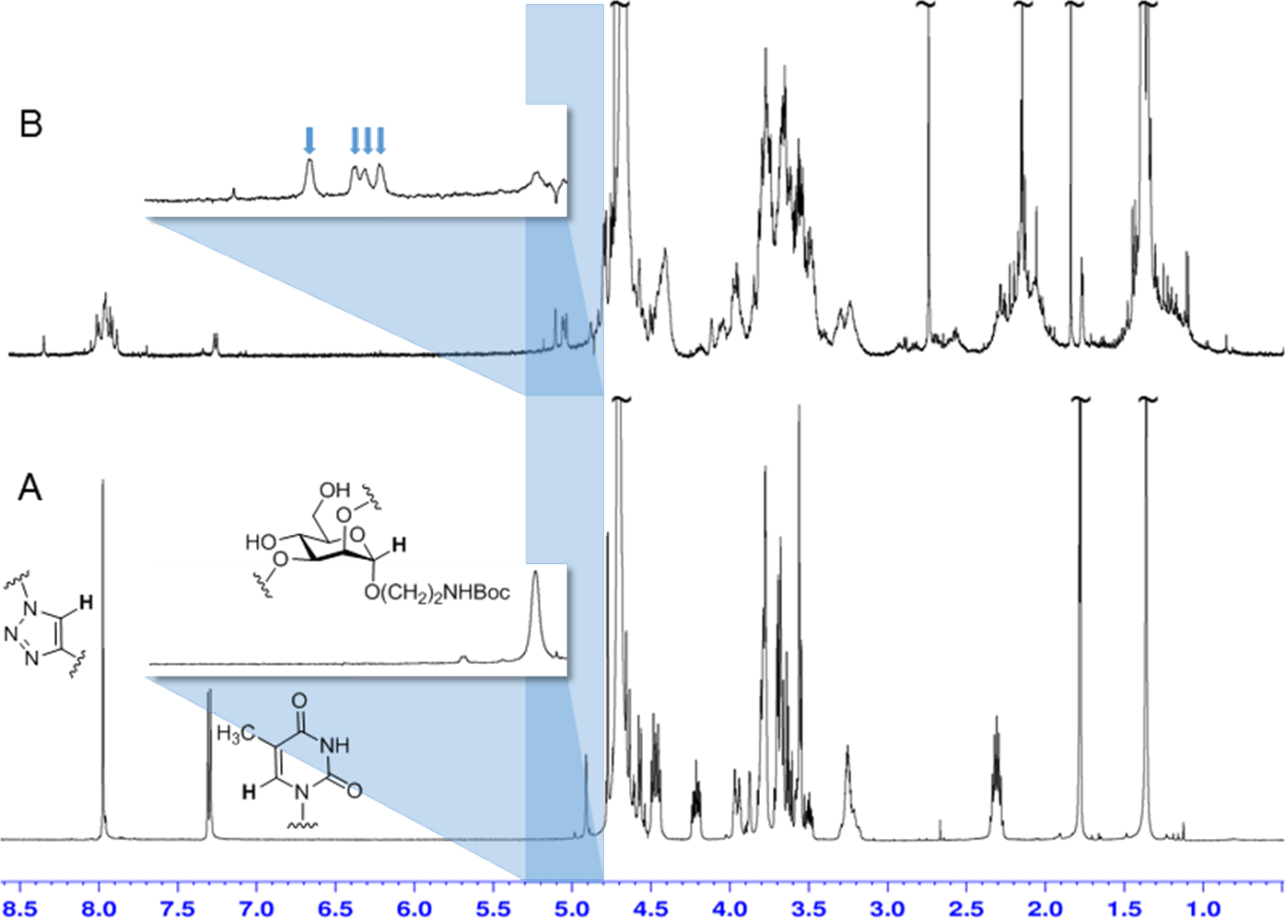
^1^H NMR spectra (all in D_2_O, 500 MHz) of mannoside **13** (A) and of the irradiation product (**14**) after 6 hours irradiation time (B). Photocycloaddition is indicated by the changes of the signals for the triazole H-11 and the thymine H-6 protons (left at low field) and the anomeric H-1 of the carbohydrate scaffold (≈4.9 ppm). The anomeric H-1 region is detailed in both cases: four new signals are seen in **14** (B) (blue arrows) corresponding to four different isomeric products of the photocycloaddition.

For photodimerization of **13**, it was irradiated for 6 h, again in diluted solution using a 1:1 mixture of water and acetone. As it was observed in the irradiation of **7**, the peaks for the thymine H-6 protons, the triazole H-11, and the anomeric H-1 of the core mannoside underwent a characteristic change in the ^1^H NMR spectrum (Figure 3). In this case, four, not five (as in **8**), new doublets for H-1_core_ appear in the photodimerized product **14** (Figure 3B). This finding is in line with the formation of two *cis–syn-* and two *trans–syn*-[2 + 2] photocycloaddition products. The formation of *anti*-addition products seems to be hampered in this case because the glycothymine branches in **13** and **14** are sterically more hindered than the thymine branches in **7** and **8**. Again UV–vis spectroscopy further underlines the dimerization success as the absorption maximum at 270 nm disappears and mass spectrometry supports intramolecular photocycloaddition only (cf. Supporting Information File 1).

## Conclusion

In line with our former work on the orientational control of carbohydrate ligands assembled on a surface [6], we seeked the synthesis of multivalent glycoconjugates that allow the organization of multivalency. To start this new approach, we have introduced a divalent carbohydrate-scaffolded glycothymine system and showed intramolecular *syn*-[2 + 2] photocycloaddition as planned. This photoreaction changes the conformational availability of the conjugated α-D-mannosyl ligands and thus adds a regulating parameter to multivalency studies. Biological assays employing this type of photocontrollable glycoconjugates will have to follow.

## Supporting Information

File 1 Experimental and analytical details and NMR spectra for all new synthetic compounds as well as discussion of [2 + 2] photocycloaddition with **7** and **13**.
