# Supplementary material for: Synthesis of carbohydrate-scaffolded thymine glycoconjugates to organize multivalency
Source: Beilstein J Org Chem. 2015 May 7;11:668–74. doi: 10.3762/bjoc.11.75 (PMC4464435; doi:10.3762/bjoc.11.75)
Supplement: File 1 — Experimental and analytical details and NMR spectra for all new synthetic compounds as well as discussion of [2 + 2] photocycloaddition with 7 and 13. [file Beilstein_J_Org_Chem-11-668-s001.pdf]

Supporting Information

for

Synthesis of carbohydrate-scaffolded thymine glycoconjugates to organize multivalency

Anna K. Ciuk and Thisbe K. Lindhorst*

Address: Christiana Albertina University of Kiel, Otto Diels Institute of Organic Chemistry, Otto-Hahn-Platz 3/4, D-24118 Kiel, Germany, Fax: +49 431 8807410

E-mail: Thisbe K. Lindhorst - tklind@oc.uni-kiel.de

*Corresponding author

Experimental and analytical details and NMR spectra for all new synthetic compounds as well as discussion of [2 + 2] photocycloaddition with 7 and 13

General methods and experimental procedures -S2-

NMR spectra -S13-

[2+2] Photocycloaddition -S24-

Materials and general methods

All purchased chemicals were used without further purification. Moisture-sensitive reactions were carried out under nitrogen in dry glassware. ^{1}H and ^{13}C NMR spectra were recorded on Bruker DRX-500 at 300 K and 500.13 and 125.75 MHz, respectively. Chemical shifts are reported relative to internal D_2O (δ = 4.76 ppm), MeOH-d_4 (^{1}H: δ = 3.31 ppm, ^{13}C: δ = 49.0 ppm), DMSO-d_6 (^{1}H: δ = 2.50 ppm, ^{13}C: δ = 39.5 ppm). Full assignment of the peaks was achieved with the aid of 2D NMR techniques (^{1}H-^{1}H COSY and ^{1}H-^{13}C HSQC). Melting points were measured on a Electrothermal Instrument and are uncorrected. ESI mass spectra were recorded on a Marina ESI-ToF 5280 from Applied Biosystems. MALDI–TOF spectra were recorded on a Biflex-III from Bruker-Daltonics (N_2-laser at 337 nm, 19 kV, Matrix: 4-CCA in acetronitrile/0.1 % TFA (1:2)). Optical rotations were measured with a Perkin-Elmer 341 polarimeter (sodium D-line: 589 nm, length of cell: 1 dm) in the indicated solvents. Thin-layer chromatography was performed on precoated silica gel plates on aluminum 60 F254 (E. Merck 5554). Detection was effected by UV and/or charring with 10% sulfuric acid in EtOH followed by heat treatment at ≈180 °C. Flash chromatography was performed on silica gel 60 (0.04–0.063 mm, 60 A, Merck) using distilled solvents. Irradiation was performed with a middle pressure mercury lamp TQ-150 (150 W) from Hassa with a Duran cooling jacket as cut-off-filter W295. IR spectra were recorded on FTIR spectrometer Paragon 1000 from Perkin Elmer with a golden gate diamond ATR unit and a sapphire stamp. UV–vis spectra were recorded on UV–vis spectrometer Lambda 14 from Perkin-Elmer (lenght of cell: 1 cm).

For NMR assignments the following numbering was used:

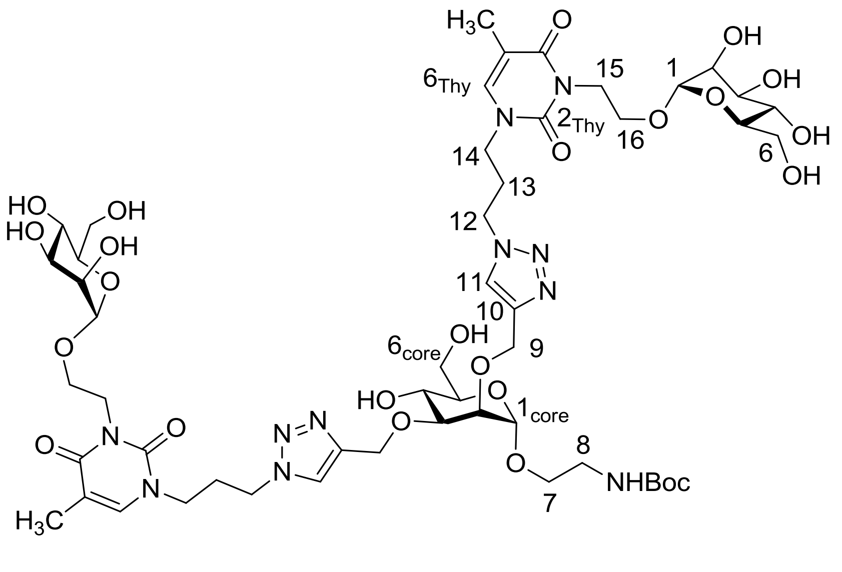

2-Azidoethyl 4,6-*O*-isopropylidene-α-D-mannopyranoside (2). A solution of mannoside **1** (1.00 g, 4.01 mmol) in dry DMF (9 mL) was treated with 2-methoxypropene (420 μL, 4.46 mmol, 1.1 equiv) and *p*-TsOH (9.00 mg, 47.3 μmol, 0.01 equiv) and the reaction mixture stirred for 2 h at rt. The reaction was quenched by addition of water (50 mL) and the aq phase was extracted with CH_2Cl_2 (4 × 50 mL). The combined organic phases were dried over $MgSO_4$, it was filtered and the solvent was removed under reduced pressure. Purification of the crude product by column chromatography (cyclohexane/ethyl acetate, 1:2) gave the mannoside **2** as a colourless solid (880 mg, 3.04 mmol, 75%). MP 82-84 °C; R_f 0.11 (cyclohexane/ethyl acetate, 1:1); $[\alpha]_D^{20}$ +38.1 (*c* 0.56, MeOH); ^{1}H NMR (500 MHz, MeOH-d_4): δ 4.83 (d, $J_{1,2}$ = 1.5 Hz, 1H, H-1), 4.03 (dd ~ t, $J_{3,4}$ = 9.7 Hz, $J_{4,5}$ = 9.7 Hz, 1H, H-4), 3.92 (dd, $J_{1,2}$ = 1.5 Hz, $J_{2,3}$ = 3.5 Hz, 1H, H-2), 3.90 (ddd, $J_{8a,7a}$ = 4.8 Hz, $J_{8b,7a}$ = 5.6 Hz, $J_{7a,7b}$ = 10.7 Hz, 1H, H-7a), 3.82 (dd, $J_{2,3}$ = 3.5 Hz, $J_{3,4}$ = 9.7 Hz, 1H, H-3), 3.86 (dd, $J_{5,6a}$ = 9.5 Hz, $J_{6a,6b}$ = 10.4 Hz, 1H, H-6a), 3.80 (dd, $J_{5,6b}$ = 5.6 Hz, $J_{6a,6b}$ = 10.4 Hz, 1H, H-6), 3.68-3.62 (m, 2H, H-7b, H-5), 3.45 (dd ~ t, $J_{7a,8}$ = 4.9 Hz, $J_{7b,8}$ = 4.9 Hz, 2H, 2 H-8), 1.56, 1.41 (each s, each 3H, $C(CH_3)_2$) ppm; ^{13}C NMR (125 MHz, MeOH-d_4): δ = 101.1 ($\underline{C}(CH_3)_2$), 102.8 (C-1), 72.5

(C-2), 72.3 (C-4), 69.8 (C-3), 67.8 (C-7), 66.3 (C-5), 63.3 (C-6), 51.7 (C-8), 29.6, 19.5 (C($\underline{C}H_3$)$_2$) ppm; IR (ATR) ṽ = 3270, 2925, 2101, 1377, 1265, 1196, 1135, 1070, 1025, 853 cm^{-1}; ESI MS $[M+Na]^+$ calcd for $C_{11}H_{19}N_3O_6$: 312.117; found *m/z* 312.113 $[M+Na]^+$.

2-Azidoethyl 4,6-*O*-isopropylidene-2,3-di-*O*-propargyl-α-D-mannopyranoside (3). A solution of mannoside **2** (450 mg, 1.56 mmol) in dry DMF (10 mL) was treated with sodium hydride (60% in mineral oil, 250 mg, 6.23 mmol) and the reaction mixture stirred for 30 min at rt. Then propargyl bromide (80% in toluene) (1.00 mL, 9.33 mmol) and TBAI (287 mg, 778 μmol) were added and the reaction was stirred overnight at rt. It was quenched by addition of water (20 mL) and the aq phase was extracted with CH_2Cl_2 (3 × 30 mL). The combined organic phases were washed with water (30 mL), dried over $MgSO_4$ and after filtration the solvent was removed under reduced pressure. Purification of the crude product by column chromatography (cyclohexane/ethyl acetate, 5:2) gave the mannoside **3** as a colourless solid (455 mg, 1.25 mmol, 80%). MP 66 °C; R_f 0.29 (cyclohexane/ethyl acetate, 5:1); $[\alpha]_D^{20}$ +35.3 (*c* 0.4, MeOH); ^{1}H NMR (500 MHz, MeOH-d_4): δ 4.99 (d, $J_{1,2}$ = 1.6 Hz, 1H, H-1), 4.45-4.30 (m, 4H, 2 $CH_{2,propargyl}$), 4.10 (dd ~ t, $J_{4,5}$ = 9.7 Hz, $J_{3,4}$ = 9.8 Hz, 1H, H-4), 4.08 (dd, $J_{1,2}$ = 1.6 Hz, $J_{2,3}$ = 3.4 Hz, 1H, H-2), 3.93 (dd, $J_{2,3}$ = 3.4 Hz, $J_{3,4}$ = 9.8 Hz, 1H, H-3), 3.93 (ddd, $J_{7a,8a}$= 4.8 Hz, $J_{7a,8b}$= 5.1 Hz, $J_{7a,7b}$= 10.7 Hz, 1H, H-7a), 3.83 (m_c, 1H, H-6a), 3.82 (m_c, 1H, H-6b), 3.70-3.63 (m, 2H, H-5, H-7a), 3.45 (m_c, 2H, 2 H-8), 2.93, 2.87 (each m_c, 2H, 2 $CH_{propargyl}$), 1.54, 1.40 (each s, each 3H, C(CH_3)$_2$) ppm; ^{13}C NMR (125 MHz, MeOH-d_4): δ 99.7 ($\underline{C}$(CH_3)$_2$), 99.2 (C-1), 79.3, 79.2 (2 $C_{propargyl}$), 75.9 (C-2), 75.5 (C-3), 74.9, 74.7 (2 $CH_{propargyl}$), 71.0 (C-4), 66.6 (C-7), 65.0 (C-5), 61.9 (C-6), 58.4, 57.6 (2 $CH_{2,propargyl}$), 50.3 (C-8), 28.2, 18.1 (C($\underline{C}H_3$)$_2$) ppm; IR (ATR) ṽ = 3263, 2924, 2878, 2103, 1497, 1451, 1373, 1266, 1199, 1110, 1084, 1059, 1021, 913, 862 cm^{-1}; ESI MS $[M+Na]^+$ calcd for $C_{17}H_{23}N_3O_6$: 388.148; found *m/z* 388.146 $[M+Na]^+$.

2-Azidoethyl 2,3-di-*O*-propargyl-α-D-mannopyranoside (4). The isopropylidene-protected mannoside **3** (300 mg, 821 μmol) was dissolved in CH_2Cl_2/TFA (9:1) (5 mL) and the reaction mixture stirred for 10 min at rt. Half-satd. aq Na_2CO_3 solution (10 mL) was added and the aq phase was extracted with CH_2Cl_2 (30 mL). The organic phases were combined and dried over $MgSO_4$. After filtration the solvent was removed under reduced pressure and the crude product purified by column chromatography (cyclohexane/ethyl acetate, 1:2) to give the title mannoside **4** as a colourless syrup (292 mg, 898 μmol, 86%.). R_f 0.24 (cyclohexane/ethyl acetate, 1:2); $[\alpha]_D^{20}$ = +38.6 (*c* 0.5, MeOH); ^{1}H NMR (500 MHz, MeOH-*d4*): δ 4.98 (d, $J_{1,2}$ = 1.8 Hz, 1H, H-1), 4.40-4.30 (m, 4H, 2 $CH_{2,propargyl}$), 4.08 (dd, $J_{1,2}$ = 1.8 Hz, $J_{2,3}$ = 3.3 Hz, 1H, H-2), 3.92 (ddd, $J_{7a,8a}$= 4.9 Hz, $J_{7a,8b}$= 5.2 Hz, $J_{7a,7b}$= 11.1 Hz, 1H, H-7a), 3.83 (dd, $J_{5,6a}$ = 2.3 Hz, $J_{6a,6b}$ = 11.9 Hz, 1H, H-6a), 3.80 (dd, $J_{2,3}$ = 3.3 Hz, $J_{3,4}$ = 9.6 Hz, 1H, H-3), 3.71-3.63 (m, 3H, H-4, H-6b, H-7b), 3.50 (ddd, $J_{5,6a}$ = 2.3 Hz, $J_{5,6b}$ = 6.1 Hz, $J_{4,5}$ = 9.3 Hz, 1H, H-5), 3.43 (m_c, 2H, 2 H-8), 3.89-3.88 (m, 1H, $CH_{propargyl}$), 3.85-3.83 (m, 1H, $CH_{propargyl}$) ppm; ^{13}C NMR (125 MHz, MeOH-*d*4): δ 99.6 (C-1), 81.0, 80.7 (2 $C_{propargyl}$), 80.3 (C-3), 76.2, 76.1 (2 $CH_{propargyl}$), 76.0 (C-2), 75.1 (C-5), 67.8 (C-7), 67.8 (C-4), 62.9 (C-6), 59.3, 58.8 (2 $CH_{2,propargyl}$), 51.7 (C-8) ppm; IR (ATR) ṽ = 3269, 2923, 2105, 1466, 1357, 1276, 1261, 1137, 1095, 1055, 1011, 764, 750 cm^{-1}; MALDI-ToF MS $[M+H]^+$ calcd. for $C_{14}H_{19}N_3O_6$: 326.1352; found *m/z* 325.9912 $[M+H]^+$.

2-*tert*-Butoxycarbonylamidoethyl 2,3-di-*O*-propargyl-α-D-mannopyranoside (5). For Staudinger reduction, a solution of mannoside **4** (182 mg, 559 μmol) in dry THF (6 mL) was treated with triphenylphospine (220 mg, 839 μmol) and the reaction mixture stirred for 3 h at rt. After addition of water (6 mL) the reaction was stirred rigorously overnight. The solvents were removed under reduced pressure and the residual crude product was taken up in dry MeOH (6 mL) and after addition of Boc_2O (183 mg, 839 μmol) the solution was stirred overnight at rt. The solvent was removed under reduced pressure and purification of the crude

product by column chromatography (CH_2Cl_2/MeOH, 20:1) gave the mannoside **5** as a colourless syrup (174 mg, 433 μmol, 78%). R_f 0.09 (CH_2Cl_2/MeOH, 15:1); $[\alpha]_D^{20}$ +37.9 (*c* 0.5, MeOH); ^{1}H NMR (500 MHz, MeOH-d_4): δ 4.92 (d, $J_{1,2}$ = 1.7 Hz, 1H, H-1), 4.40-4.30 (m, 4H, 2 $CH_{2,propargyl}$), 4.03 (dd, $J_{1,2}$ = 1.7 Hz, $J_{2,3}$ = 3.1 Hz, 1H, H-2), 3.81 (dd, $J_{5,6a}$ = 2.3 Hz, $J_{6a,6b}$ = 11.8 Hz, 1H, H-6a), 3.76 (dd, $J_{2,3}$ = 3.1 Hz, $J_{3,4}$ = 9.5 Hz, 1H, H-3), 3.75-3.65 (m, 3H, H-4, H-6b, H-7a), 3.56-3.46 (m, 2H, H-5, H-7b), 3.27-3.19 (m, 2H, 2 H-8), 2.88-2.85 (m, 2H, 2 $CH_{propargyl}$), 1.45 (s, 9H, Boc-$C(CH_3)_3$) ppm; ^{13}C NMR (125 MHz, MeOH-d_4): δ 158.5 (Boc-C(O), 99.3 (C-1), 81.0, 80.8 (2 $C_{propargyl}$), 80.5 (C-3), 80.2 (Boc-$\underline{C}(CH_3)_3$), 76.1, 76.0 (2 $CH_{propargyl}$), 76.0 (C-2), 74.9 (C-5), 67.8 (C-7), 67.7 (C-4), 62.8 (C-6), 59.2, 58.7 (2 $CH_{2,propargyl}$), 41.1 (C-8), 28.8 (Boc-$C(\underline{C}H_3)_3$) ppm; IR (ATR): $\tilde{\nu}$ = 3391, 3293, 2935, 1695, 1531, 1367, 1174, 1137, 1111, 1062, 1034, 723 cm^{-1}; MALDI-ToF MS $[M+Na]^+$ calcd. for $C_{17}H_{23}N_3O_6$: 422.11791; found *m/z* 421.9698 $[M+Na]^+$.

2-*tert*-Butoxycarbonylamidoethyl 2,3-di-*O*-[4-(thymin-N^1-ylpropyl)-triazolylmethyl]-α-D-mannopyranoside (7). Mannoside **5** (70.0 mg, 175 μmol) and N^1-(3-azidopropyl)thymine (**6**, 81.0 mg, 386 μmol) were dissolved in DMF/water (4 mL:1.2 mL) and the mixture was treated with a solution of $CuSO_4 \cdot 5\,H_2O$ (18.0 mg, 70.1 μmol) in water (100 μL) and sodium ascorbate (28.0 mg, 140 μmol). The reaction mixture was stirred overnight at rt and then it was filtered over celite and the solvents were removed under reduced pressure. Purification of the crude product by column chromatography (CH_2Cl_2/MeOH, 5:1) and following lyophilisation gave the title di-thymine glycoconjugate **7** as a colourless lyophilisate (102 mg, 125 μmol, 71%); R_f 0.33 (CH_2Cl_2 / MeOH, 10 : 1); $[\alpha]_D^{20}$ +3.8 (*c* 0.5, H_2O); ^{1}H NMR (500 MHz, D_2O): δ 7.96 (s, 2H, 2 H-11), 7.28-7.24 (m, 2H, 2 H-6_{Thy}), 4.92 (br s, 1H, H-1), 4.68-4.54 (m, 4H, 4 H-9), 4.50-4.43 (m, 4H, 4 H-14), 3.86 (br s, 1H, H-2), 3.80-3.60 (m, 9H, H-3, H-4, H-6a, H-6b, H-7a, 4 H-12), 3.60-3.54 (m, 1H, H-5), 3.52-3.46 (m, 1H, H-7b), 3.28-3.16 (m, 2H, 2 H-8), 2.34-2.26 (m, 4H, 4 H-13), 1.74, 1.73 (each d, 6H,

J_{6,CH_3} = 1.0 Hz, 2 $CH_{3,Thy}$), 1.35 (s, 9H, Boc-$C(CH_3)_3$) ppm; ^{13}C NMR (125 MHz, D_2O): δ 166.7 (2 C-4_{Thy}), 158.2 (Boc-C(O)), 152.0 (2 C-2_{Thy}), 144.2, 144.1 (2 C-10), 142.5 (2 C-6_{Thy}), 125.0, 124.7 (2 C-11), 110.6 (2 C-5_{Thy}), 97.1 (C-1), 80.9 (Boc-$\underline{C}(CH_3)_3$), 78.4 (C-3), 74.4 (C-2), 72.8 (C-5), 66.5 (C-7), 65.9 (C-4), 63.0, 62.4 (2 C-9) 60.8 (C-6), 48.1 (2 C-14), 46.2 (2 C-12), 39.6 (C-8), 27.8, 27.7 (C-13), 27.7 (Boc-$C(\underline{C}H_3)_3$), 11.2 (2 $CH_{3,Thy}$) ppm; IR (ATR): $\tilde{\nu}$ = 3363, 2973, 1668, 1469, 1365, 1276, 1257, 1121, 1055, 1033, 765 cm^{-1}; MALDI-ToF MS $[M+Na]^+$ calcd. for $C_{35}H_{51}N_{11}O_{12}$: 840.3616; found *m/z* 840.6373 $[M+Na]^+$.

Irradiation of 7: [2 + 2] photocycloaddition to 8. The di-thymine glycoconjugate **7** (20.0 mg, 25.3 µmol) was dissolved in water/acetone (1:1) (40 mL) and degassed with nitrogen for 30 min. Then the solution was irradiated in a quarz glass flask for 6 h. After removal of the solvents under reduced pressure the following lyophilisation gave **8** (20 mg) as an isomeric mixture of products and ≈7% remaining starting material (**7**, according to integral ratios in the ^1H NMR). Spectroscopic characterization was performed without further treatment or purification, respectively; MALDI-ToF MS $[M+Na]^+$ calcd. for $C_{35}H_{51}N_{11}O_{12}$: 840.3616; found *m/z* 840.5980 $[M+Na]^+$; mass peaks corresponding to intermolecular photocycloaddition products were not detected.

2-*tert*-Butoxycarbonylamidoethyl 4,6-*O*-isopropylidene-2,3-di-*O*-propargyl-α-D-mannopyranoside (9). For Staudinger reduction, a solution of mannoside **3** (573 mg, 1.57 mmol) in dry THF (12 mL) was treated with triphenylphosphine (616 mg, 2.35 mmol) and the reaction mixture stirred at rt for 3 h. After addition of water (12 mL) the reaction was stirred rigorously overnight. The solvents were removed under reduced pressure and the crude product was taken up in dry MeOH (12 mL) and after addition of Boc_2O (514 mg, 2.35 mmol) the reaction mixture was stirred overnight at rt. The solvent was removed under reduced pressure and purification of the crude product by column chromatography (cyclohexane/ethyl acetate, 2:1) gave the title mannoside **9** as a colourless syrup (599 mg, 1.36 mmol, 87%). R_f

0.36 (cyclohexane / ethyl acetate, 2 : 1); $[\alpha]_D^{20}$ +40.3 (*c* 0.4, MeOH); ^{1}H NMR (500 MHz, MeOH-d_4): δ = 4.89 (d, $J_{1,2}$ = 1.6 Hz, 1H, H-1), 4.40-4.27 (m, 4H, 2 $CH_{2,propargyl}$), 4.04 (dd ~ t, $J_{3,4}$ = 9.8 Hz, $J_{4,5}$ = 9.9 Hz, 1H, H-4), 4.02 (dd, $J_{1,2}$ = 1.6 Hz, $J_{2,3}$ = 3.3 Hz, 1H, H-2), 3.87 (dd, $J_{2,3}$ = 3.3 Hz, $J_{3,4}$ = 9.8 Hz, 1H, H-3), 3.81-3.76 (m, 2H, H-6a, H-6b), 3.69 (ddd, $J_{7a,8a}$ = 4.7 Hz, $J_{7a,8b}$ = 7.4 Hz, $J_{7a,7b}$ = 10.1 Hz, 1H, H-7a), 3.61 (ddd~ td, $J_{5,6a}$ = 6.6 Hz, $J_{5,6b}$ = 9.4 Hz, $J_{4,5}$ = 9.9 Hz, 1H, H-5), 3.47 (ddd, $J_{7b,8a}$ = 5.0 Hz, $J_{7b,8b}$ = 5.0 Hz, $J_{7a,7b}$ = 10.0 Hz, 1H, H-7b), 3.40-3.42 (m, 2H, 2 H-8), 2.90-2.88 (m, 1H, $CH_{propargyl}$), 2.87-2.85 (m, 1H, $CH_{propargyl}$), 1.51 (s, 3H, C(CH_3)), 1.46 (s, 9H, Boc-C(CH_3)$_3$), 1.36 (s, 3H, C(CH_3)) ppm; ^{13}C NMR (125 MHz, MeOH-d_4): δ = 158.6 (Boc-C(O)), 101.0 (C-1), 100.3 ($\underline{C}(CH_3)_2$), 80.9, 80.7 (2 $C_{propargyl}$), 80.2 (Boc-$\underline{C}(CH_3)_3$), 77.9, 77.7 (2 $CH_{propargyl}$), 76.2 (C-2), 75.2 (C-3), 72.5 (C-4), 67.7 (C-7), 66.1 (C-5), 63.3 (C-6), 59.7, 59.0 (2 $CH_{2,propargyl}$), 41.1 (C-8), 29.6 (C($\underline{C}H_3$)), 28.7 (Boc-C($\underline{C}H_3$)$_3$), 19.6 (C($\underline{C}H_3$)) ppm; IR (ATR) ṽ 3265, 2978, 2931, 1697, 1513, 1366, 1266, 1168, 1110, 1059, 1032, 863, 751, cm^{-1}; MALDI-ToF MS [M+H]$^+$ calcd. for $C_{22}H_{33}NO_8$: 440.2284; found *m/z* 440.0188 [M+H]$^+$.

2-*tert*-Butoxycarbonylamidoethyl 4,6-*O*-isopropylidene-2,3-di-*O*-[4-(thymin-*N*^{1}-ylpropyl)-triazolylmethyl]-α-D-mannopyranoside (10). Mannoside **9** (325 mg, 739 µmol) and 1-(3-azidopropyl)thymine (**6**, 81.0 mg, 386 µmol) were dissolved in DMF/water (14 mL:4.2 mL) and treated with a solution of $CuSO_4 \cdot 5H_2O$ (74.0 mg, 296 µmol) in water (100 µL) and sodium ascorbate (117 mg, 592 µmol) and the reaction mixture was stirred overnight at rt. The reaction was quenched by addition of half-satd. aq NH_4Cl solution (20 mL) and the aq phase extracted with CH_2Cl_2 (3 × 30 mL). The combined organic phases were dried over $MgSO_4$ and after filtration the solvent was removed under reduced pressure. Purification of the crude product by column chromatography (CH_2Cl_2/MeOH, 15:1 → 10:1) gave the di-thymine mannoside **10** as a colourless solid (487 mg, 568 µmol, 77%); MP 137 °C; R_f 0.12 (CH_2Cl_2 / MeOH, 15 : 1); $[\alpha]_D^{20}$ +3.9 (*c* 0.5, DMSO); ^{1}H NMR (500 MHz, DMSO-

d_6): δ 11.21 (br s, 2H, 2 NH_{Thy}), 8.12, 8.07 (each s, 1H, 2 H-11), 7.47-7.46 (m_c, 2H, H-6_{Thy}), 6.91 (t, $J_{CH_2,NH}$ = 5.7 Hz, 1H, Boc-NH), 4.80 (d, $J_{1,2}$ = 1.1 Hz, 1H, H-1), 4.70 (d, $J_{9a,9b}$ = 12.5 Hz, 1H, H-9a), 4.66 (d, $J_{9a,9b}$ = 12.5 Hz, 1H, H-9b), 4.62 (d, $J_{9a,9b}$ = 12.1 Hz, 1H, H-9a), 4.55 (d, $J_{9a,9b}$ = 12.1 Hz, 1H, H-9b), 4.38 (m_c, 4H, 4 H-12), 3.92 (dd ~ t, $J_{3,4}$ = 9.7 Hz, $J_{4,5}$ = 9.7 Hz, 1H, H-4), 3.72-3.65 (m, 7H, H-3, H-6a, H-6b, 4 H-14), 3.55 - 3.46 (m, 2H, H-5, H-7a), 3.37-3.33 (m, 1H, H-7b), 3.17-3.30 (m, 2H, 2 H-8), 2.15 (m_c, 4H, 4 H-13), 1.73 (d, J_{6,CH_3} = 1.1 Hz, 6H, 2 $CH_{3,Thy}$), 1.42 (s, 3H, $C(CH_3)_2$), 1.38 (s, 9H, Boc-$C(CH_3)_3$), 1.31 (s, 3H, $C(CH_3)_2$) ppm; ^{13}C NMR (125 MHz, DMSO-d_6): δ 164.8 (2 C-4_{Thy}), 156.1 (Boc-C(O)), 151.4 (2 C-2_{Thy}), 144.8, 144.6 (C-10), 141.6 (2 C-6_{Thy}), 124.5, 124.2 (C-11), 109.1 (C-5_{Thy}), 99.5 ($\underline{C}(CH_3)_2$), 98.4 (C-1), 78.1 (Boc-$\underline{C}(CH_3)_3$), 76.3 (C-2), 76.1 (C-3), 71.07 (C-4), 66.6 (C-7), 64.9 (C-5), 64.5, 63.4 (C-9), 61.9 (C-6), 47.3 (2 C-14), 45.3 (2 C-12), 39.6 (C-8), 29.9 ($C(\underline{C}H_3)$), 29.6 (2 C-13), 28.7 (Boc-$C(\underline{C}H_3)_3$), 19.7 ($C(\underline{C}H_3)$), 12.4 (2 $CH_{3,Thy}$) ppm; IR (ATR) ṽ = 2934, 1668, 1463, 1365, 1116, 1048, 865, 762 cm^{-1}; MALDI-ToF MS $[M+Na]^+$ calcd. for $C_{38}H_{55}N_{11}O_{12}$: 880.3929; found *m/z* 880.7635 $[M+Na]^+$.

2-*tert*-Butoxycarbonylamidoethyl 4,6-*O*-isopropylidene-2,3-di-*O*-{4-[*N*^{3}-(2-(2,3,4,6-tetra-*O*-acetyl-α-D-mannopyranosyloxy)ethyl]thymin-*N*^{1}-ylpropyl]triazolylmethyl}-α-D-mannopyranoside (12). The di-thymine glycoconjugate **10** (200 mg, 233 μmol) and 2-bromoethyl 2,3,4,6-tetra-*O*-acetyl-α-D-mannopyranoside (**11**, 318 mg, 699 μmol) were dissolved in dry DMF (10 mL) and treated with DBU (70.0 μL, 466 μmol) and TBAI (172 mg, 466 μmol) and the reaction mixture was stirred for 2 d at rt. The solvent was removed under reduced pressure and the crude product was taken up in CH_2Cl_2 (50 mL) and washed with satd. aq NH_4Cl solution (2 × 20 mL) and water (20 mL). The organic phase was dried over $MgSO_4$ and after filtration the solvent was removed under reduced pressure. Purification of the crude product by column chromatography (CH_2Cl_2/MeOH, 20:1) gave the divalent glycothymine mannoside **12** as a colourless solid (235 mg, 146 μmol, 64%); MP 111

°C; R_f 0.12 (CH_2Cl_2/MeOH, 20:1); $[\alpha]_D^{20}$ +33.3 (*c* 0.5, DMSO); ^{1}H NMR (500 MHz, DMSO-d_6): δ 8.09, 8.05 (each s, 2H, 2 H-11), 7.56 (m_c, 2H, H-6_{Thy}), 6.90 (t, $J_{CH_2,NH}$ = 5.8 Hz, 1H, Boc-NH), 5.07-5.00 (m, 6H, H-2, H-2', H-3, H-3', H-4, H-4'), 4.88 (br s, 2H, 2 H-1), 4.79 (br s, 1H, H-1_{core}), 4.71 (d, $J_{9a,9b}$ = 12.5 Hz, 1H, H-9a), 4.66 (d, $J_{9a,9b}$ = 12.5 Hz, 1H, H-9b), 4.73 (d, $J_{9a,9b}$ = 12.1 Hz, 1H, H-9a), 4.56 (d, $J_{9a,9b}$ = 12.1 Hz, 1H, H-9b), 4.39 (m_c, 4H, 4 H-12), 4.17 (m_c, 2H, 2 H-15a), 4.09 (dd, $J_{5,6a}$ = 5.5 Hz, $J_{6a,6b}$ = 12.2 Hz, 2H, 2 H-6a), 4.00-3.95 (m, 4H, 2 H-6b, 2 H-15b), 3.92 (dd ~ t, $J_{3,4}$ = 9.8 Hz, $J_{4,5}$ = 9.8 Hz, 1H, H-4_{core}), 3.87 (br s, 1H, H-2_{core}), 3.80-3.63 (m, 13H, H-3_{core}, H-$6a_{core}$, H-$6b_{core}$, 2 H-5, 4 H-16, 4 H-14), 3.56-3.47 (m, 2H, H-7a, H-5_{core}), 3.39-3.30 (m, 1H, H-7b), 3.15-3.05 (m, 2H, 2 H-8), 2.18 (m_c, 4H, 4 H-13), 2.09, 2.01, 2.00, 1.90 (each s, each 6H, 8 C(O)CH_3), 1.79 (d, J_{6,CH_3} = 0.9 Hz, 6H, 2 $CH_{3,Thy}$), 1.42 (s, 3H, C(CH_3)), 1.38 (s, 9H, C$(CH_3)_3$), 1.38 (s, 3H, C(CH_3)) ppm; ^{13}C NMR (125 MHz, DMSO-d_6): δ 170.5 (2 C(O)CH_3), 170.0 (2 C(O)CH_3), 169.9 (4 C(O)CH_3), 163.4 (2 C-4_{Thy}), 156.1 (Boc-C(O)), 151.4 (2 C-2_{Thy}), 144.7, 144.6 (2 C-10), 140.4 (2 C-6_{Thy}), 124.4, 124.2 (2 C-11), 108.4 (2 C-5_{Thy}), 99.5 (C$(CH_3)_2$), 98.5 (C-1_{core}), 96.4 (2 C-1), 78.1 (Boc-C$(CH_3)_3$), 76.2 (C-2_{core}), 76.2 (C-3_{core}), 71.1 (C-4_{core}), 69.0 (2 C-2), 69.0 (2 C-3), 68.4 (2 C-5), 66.2 (C-7), 65.8 (2 C-4), 64.9 (C-5_{core}), 64.6 (C-9), 63.8 (2 C-16), 63.3 (C-9), 62.3 (C-6a), 62.0 (C-6_{core}), 47.3 (2 C-12), 46.6 (2 C-14), 39.1 (C-8), 39.5 (2 C-15), 29.6 (C(CH_3)), 29.4 (2 C-13), 28.7 (Boc-C$(CH_3)_3$), 21.0, 20.9, 20.9, 20.8 (8 C(O)CH_3), 19.7 (C(CH_3)), 13.0 (2 $CH_{3,Thy}$) ppm; IR (ATR) $\tilde{\nu}$ = 2935, 1743, 1698, 1641, 1464, 1367, 1218, 1133, 1046, 1034, 767, 751 cm^{-1}; MALDI-ToF MS $[M+Na]^+$ calcd. for $C_{70}H_{99}N_{11}O_{32}$: 1628.6355; found *m/z* 1629.6041 $[M+Na]^+$.

2-*tert*-Butoxycarbonylamidoethyl 2,3-di-*O*-{4-[N^3-(2-(α-D-mannopyranosyloxy)ethyl]-thymin-N^1-ylpropyl]triazolylmethyl}-α-D-mannopyranoside (13). A solution of the protected divalent cluster mannoside **12** (200 mg, 125 µmol) in dry MeOH (20 mL) and dry CH_2Cl_2 (1 mL) was treated with freshly prepared 1 M NaOMe solution (250 µL) and the

reaction mixture stirred overnight at rt. Then it was neutralized with Amberlite IR 120 ion exchange resin, filtered, washed with methanol and the solvent removed under reduced pressure. Following lyophilisation gave the deprotected glycothymine cluster **13** as a colourless lyophilisate (122 mg, 98.8 μmol, 79%); R_f 0.07 (CH_2Cl_2/MeOH, 5:1); $[\alpha]_D^{20}$ +38.1 (*c* 0.5, MeOH); ^{1}H NMR (500 MHz, MeOH-d_4): δ = 8.10, 8.08 (s, 2H, 2 H-11), 7.44 (m_c, 1H, H-6_{Thy}), 7.43 (m_c, 1H, H-6_{Thy}), 4.94 (d, $J_{1,2}$ = 1.5 Hz, 1H, H-1_{core}), 4.82-4.75 (m, 5H, 2 H-1, 2 H-9a, H-9b), 4.73 (d, $J_{9a,9b}$ = 12.2 Hz, 1H, H-9b), 4.52 (m_c, 4H, 4 H-12), 4.32 (m_c, 2H, 2 H-15a), 4.08 (m_c, 2H, 2 H-15b), 3.94-3.92 (m, 1H, H-2_{core}), 3.80-3.63 (m, 2H, 2 H-16a), 3.88-3.82 (m, 5H, H-6a, 4 H-14), 3.81-3.67 (m, 12H, H-4_{core}, H-3_{core}, H-$6a_{core}$, H-$6b_{core}$, 2 H-2, H-6a, 2 H-6b, H-7a, 2 H-16b), 3.65-3.61 (m, 4H, 2 H-3, 2 H-4), 3.60-3.56 (m, 1H, H-5_{core}), 3.54-3.50 (m, 1H, H-7b), 3.42-3.38 (m, 2H, 2 H-5), 3.35-3.30 (m, 1H, H-8a), 3.26-3.20 (m, 1H, H-8b), 2.36 (m_c, 4H, 4 H-13), 1.91 (m_c, 6H, 2 $CH_{3,Thy}$), 1.38 (s, 9H, $C(CH_3)_3$) ppm; ^{13}C NMR (125 MHz, MeOH-d_4): δ = 165.7 (2 C-4_{Thy}), 158.5 (Boc-C(O)), 153.0 (2 C-2_{Thy}), 146.4 (2 C-10), 141.4 (2 C-6_{Thy}), 125.5, 125.3 (C-11), 110.6 (C-5_{Thy}), 100.9 (2 C-1), 99.2 (C-1_{core}), 81.0 (C-3_{core}), 80.1 (Boc-$\underline{C}(CH_3)_3$), 76.7 (C-2_{core}), 74.9 (C-5_{core}), 74.8 (2 C-5), 72.5 (2 C-3), 72.0 (2 C-2), 68.5 (2 C-4), 67.8 (C-4_{core}), 67.7 (C-7), 65.1 (C-9), 64.4 (2 C-16), 62.9 (2 C-6, C-9), 62.8 (C-6_{core}), 48.3 (C-12), 48.0 (2 C-14), 41.4 (2 C-15), 41.2 (C-8), 30.4 (2 C-13), 28.8 (Boc-$C(\underline{C}H_3)_3$), 13.0 (2 $CH_{3,Thy}$) ppm; IR (ATR) $\tilde{\nu}$ = 3366, 2927, 1693, 1661, 1627, 1465, 1366, 1254, 1132, 1054, 768 cm^{-1}; MALDI-ToF MS $[M+Na]^+$ calcd. for $C_{51}H_{79}N_{11}O_{24}$: 1252.5197; found *m/z* 1252.7432 $[M+Na]^+$.

Irradiation of 13: [2 + 2] photocycloaddition to 14. The divalent glycothymine mannoside **13** (21.0 mg, 17.1 μmol) was dissolved in water/acetone (1:1) (50 mL) and was degassed with nitrogen for 30 min. The solution was irradiated in a quarz glass flask for 6 h. After the solvents were removed under reduced pressure the following lyophilisation gave the title compound **14** (21 mg) as an isomeric mixture of products and ≈14% remaining starting

material (**13**, according to integral ratios in the ^{1}H NMR). Spectroscopic characterization was performed without further treatment or purification, respectively; MALDI-ToF MS $[M+Na]^+$ calcd. for $C_{51}H_{79}N_{11}O_{24}$: 1252.5197; found *m/z* 1252.7281 $[M+Na]^+$; mass peaks corresponding to intermolecular photocycloaddition products were not detected.

NMR spectra of **2**

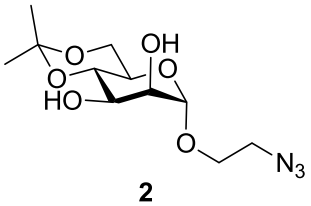

2

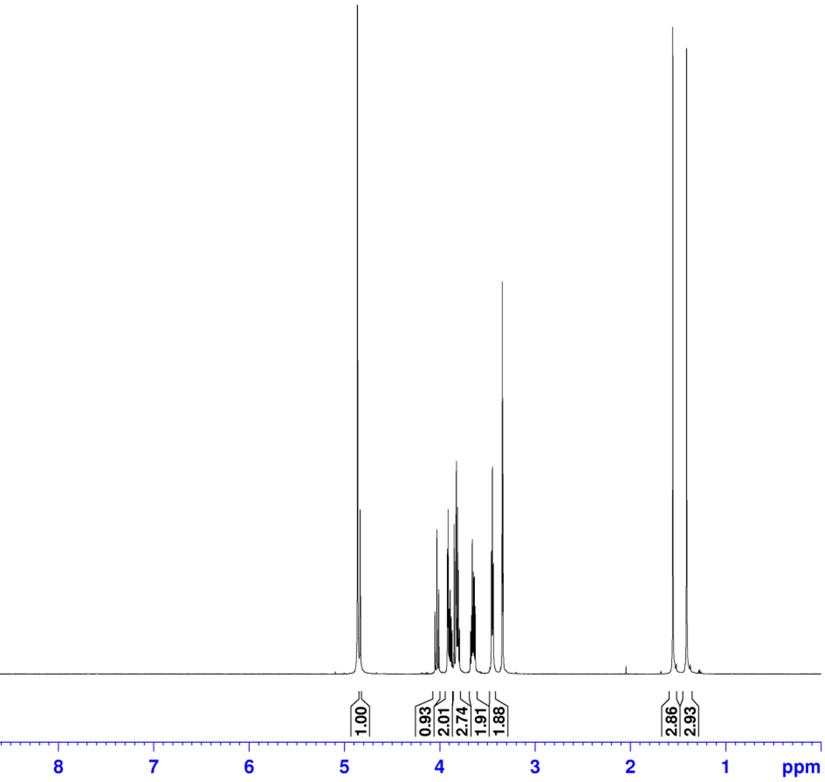

Figure S1: ^{1}H NMR spectrum (500 MHz, MeOH-d_4).

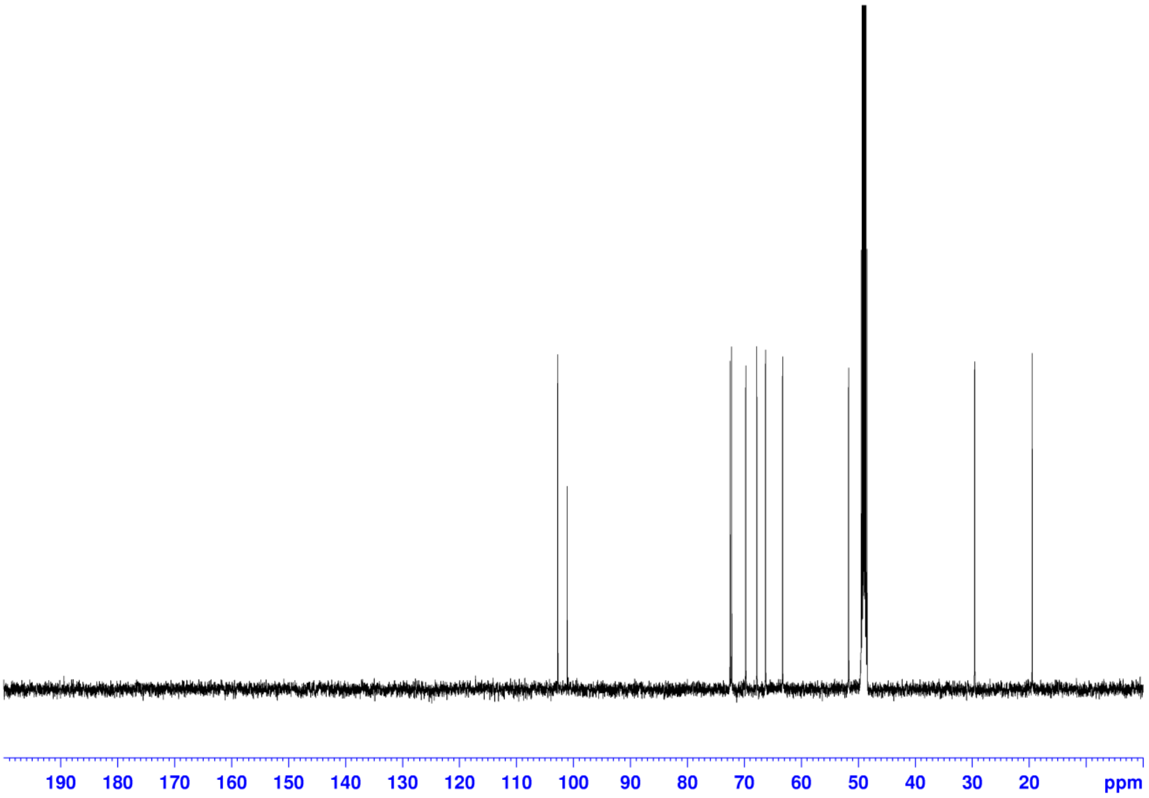

Figure S2: ^{13}C NMR spectrum (125 MHz, MeOH-d_4).

NMR spectra of **3**

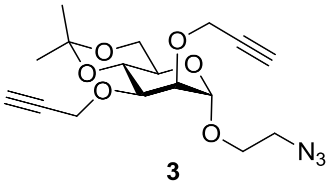

3

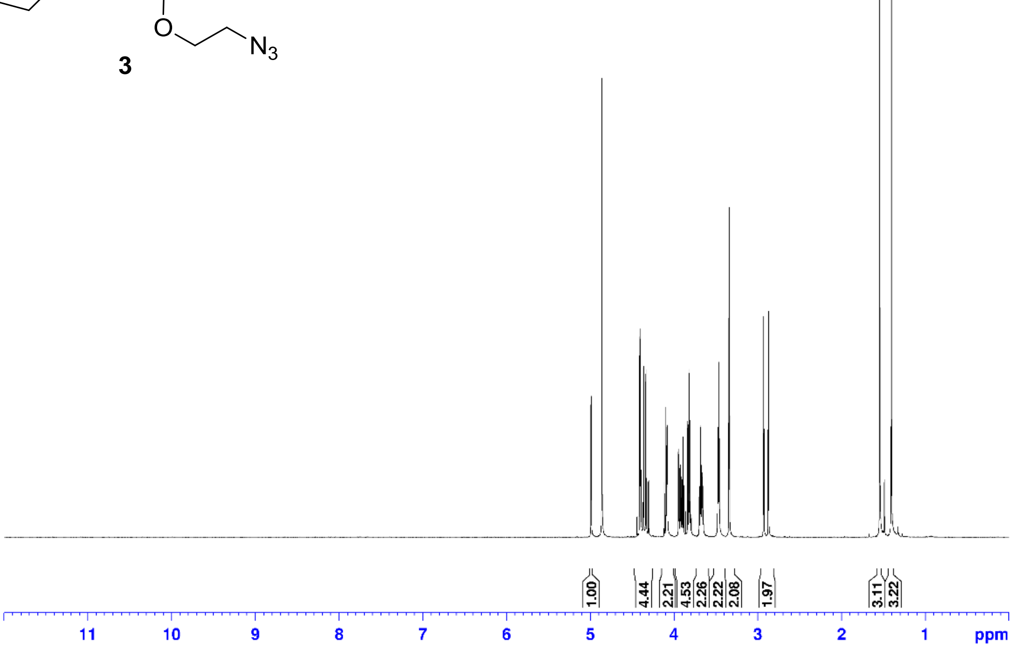

Figure S3: ^{1}H NMR spectrum (500 MHz, MeOH-d_4).

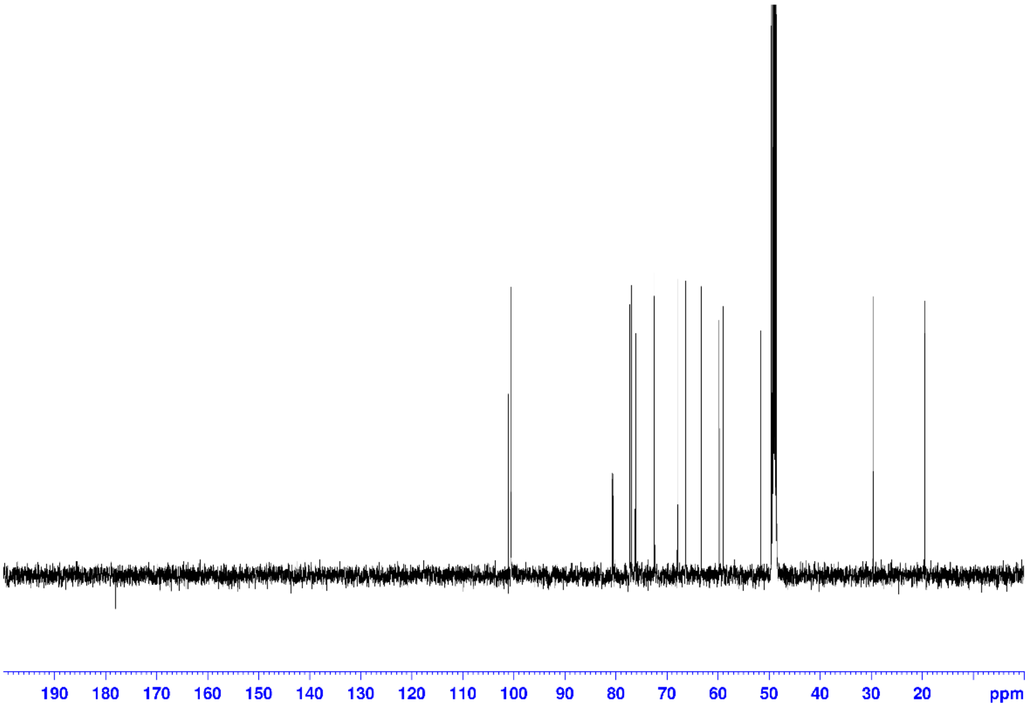

Figure S4: ^{13}C NMR spectrum (125 MHz, MeOH-d_4).

NMR spectra of **4**

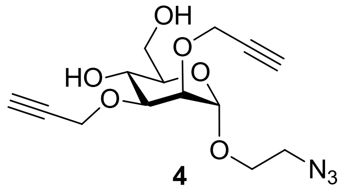

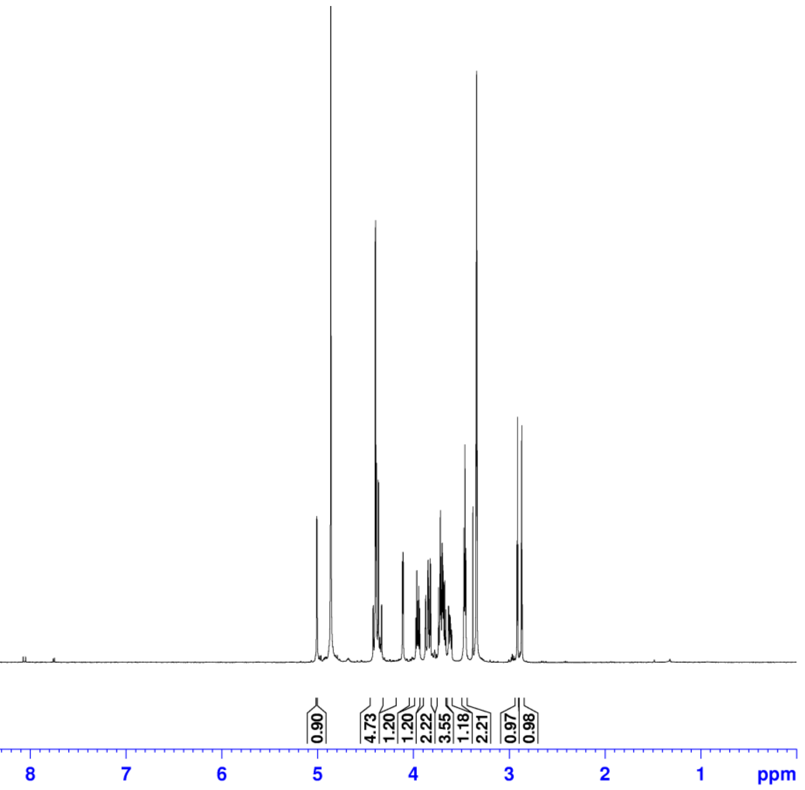

Figure S5: ^{1}H NMR spectrum (500 MHz, MeOH-d_4).

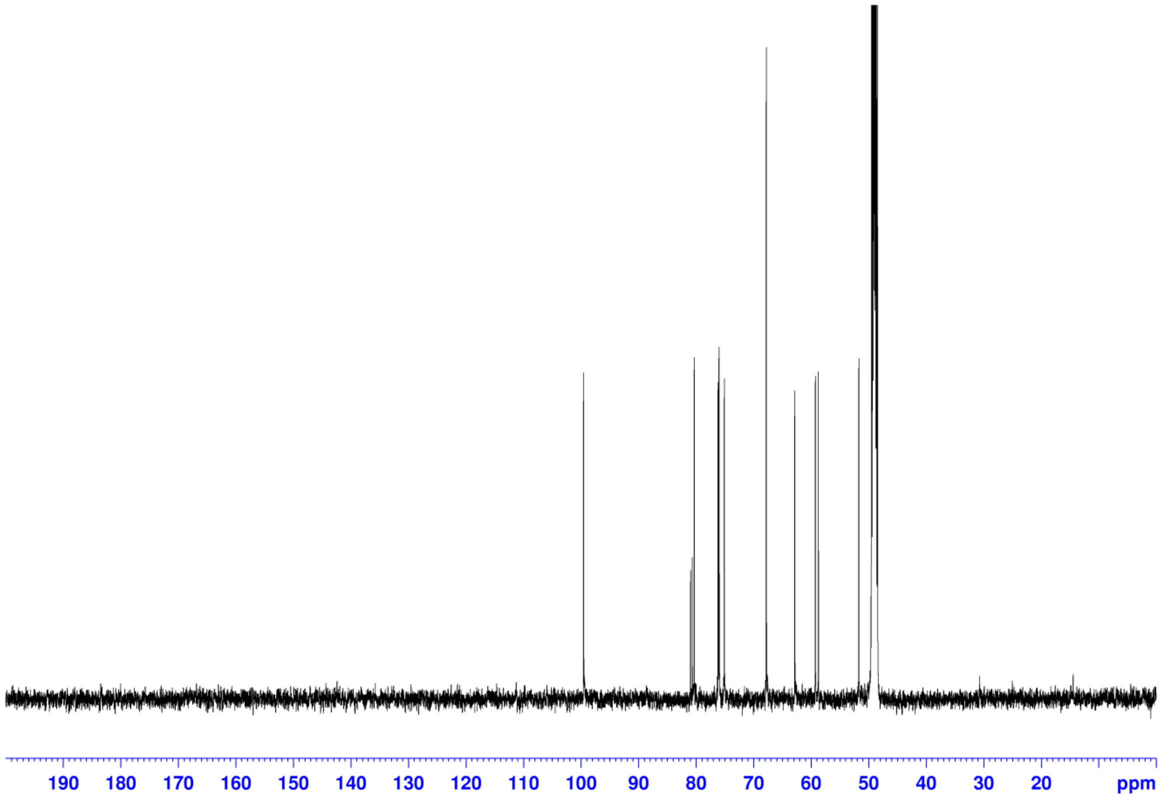

Figure S6: ^{13}C NMR spectrum (125 MHz, MeOH-d_4).

NMR spectra of **5**

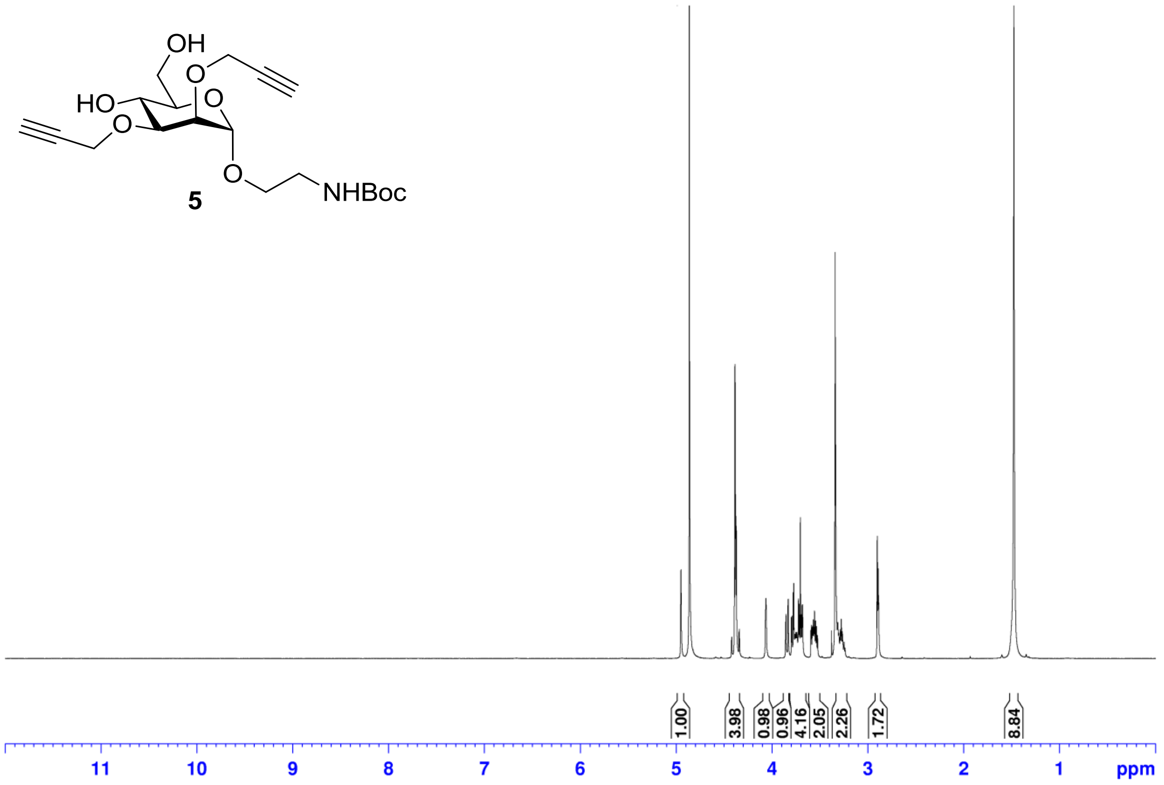

Figure S7: ^{1}H NMR spectrum (500 MHz, MeOH-d_4).

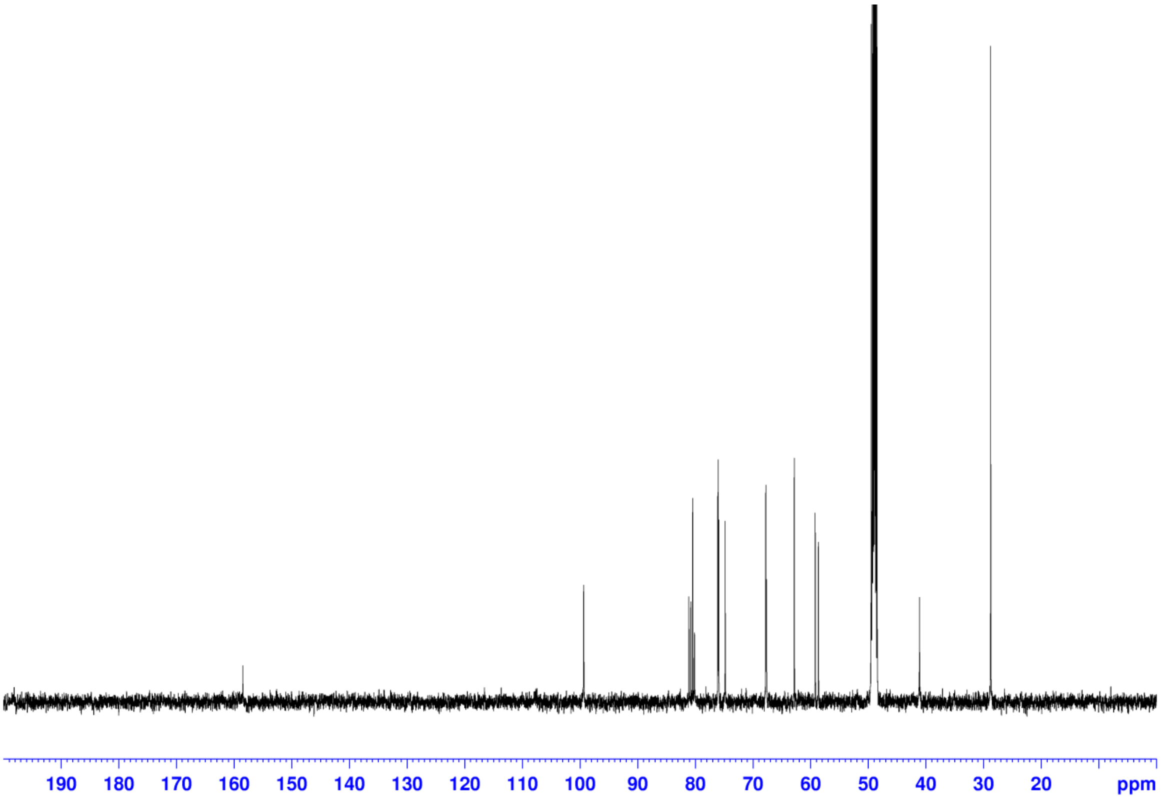

Figure S8: ^{13}C NMR spectrum (125 MHz, MeOH-d_4).

NMR spectra of **7**

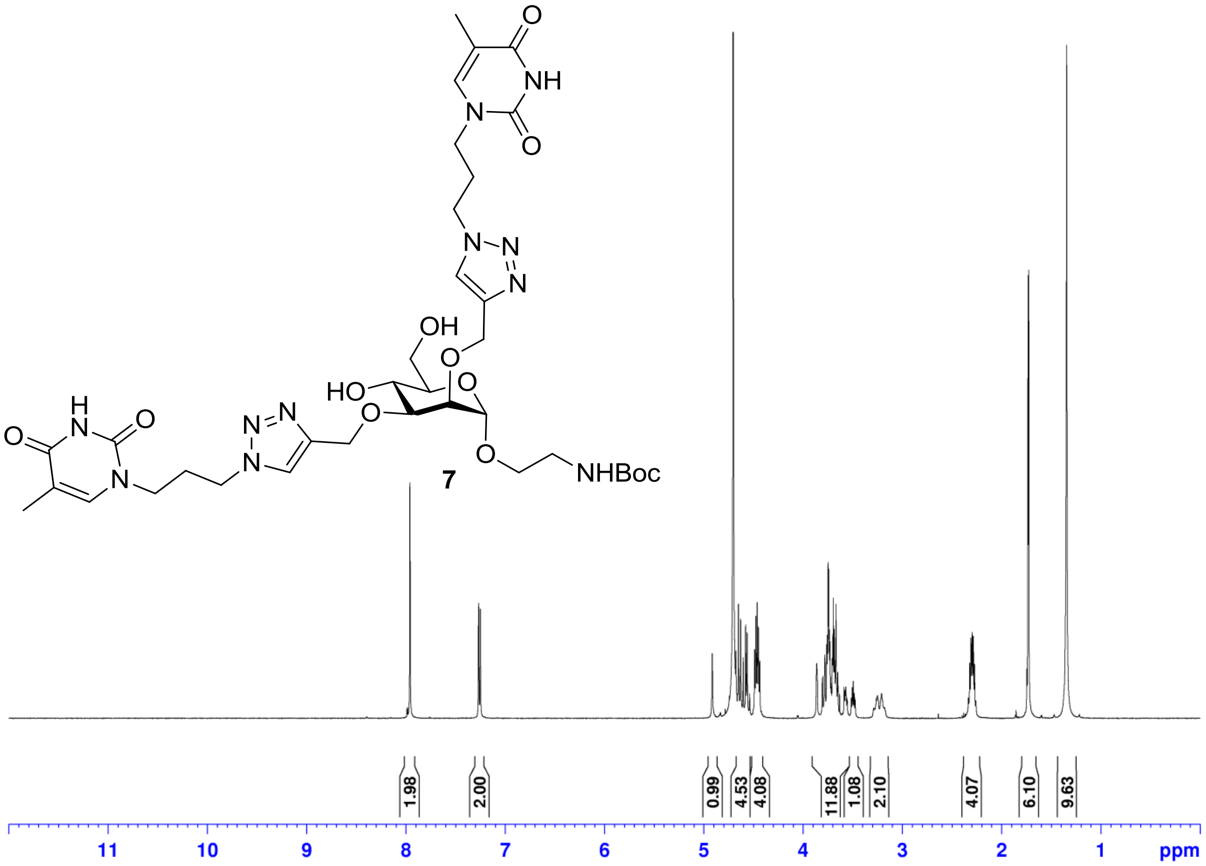

Figure S9: ^{1}H NMR spectrum (500 MHz, D_2O).

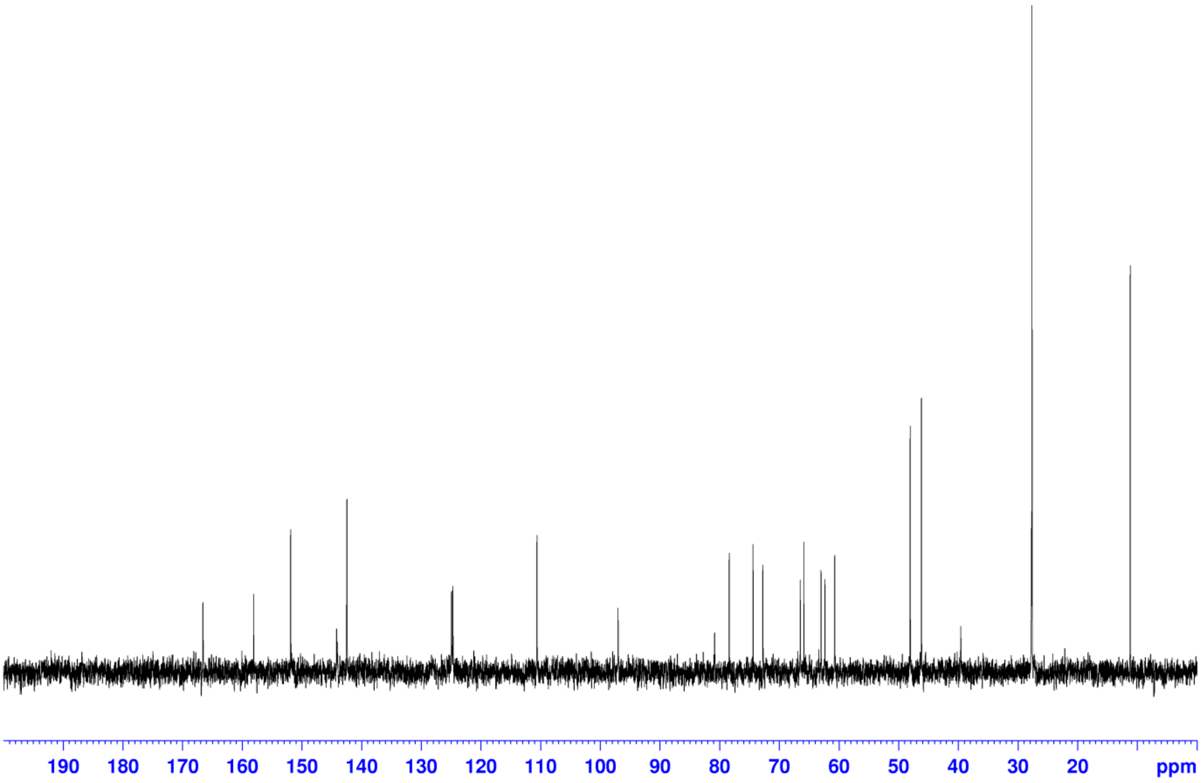

Figure S10: ^{13}C NMR spectrum (125 MHz, D_2O).

NMR spectra of **8**

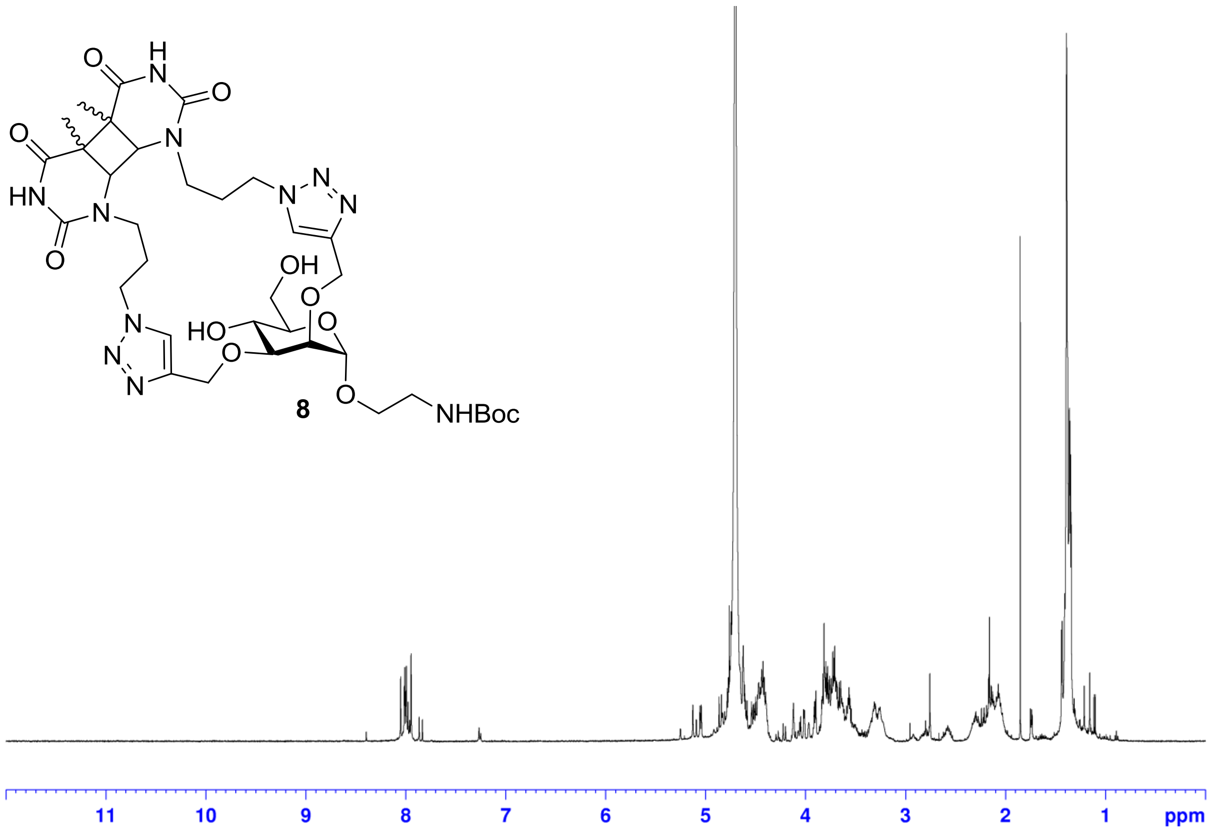

Figure S11: ^{1}H NMR spectrum of crude **8** (isomeric mixture) (500 MHz, D_2O).

NMR spectra of **9**

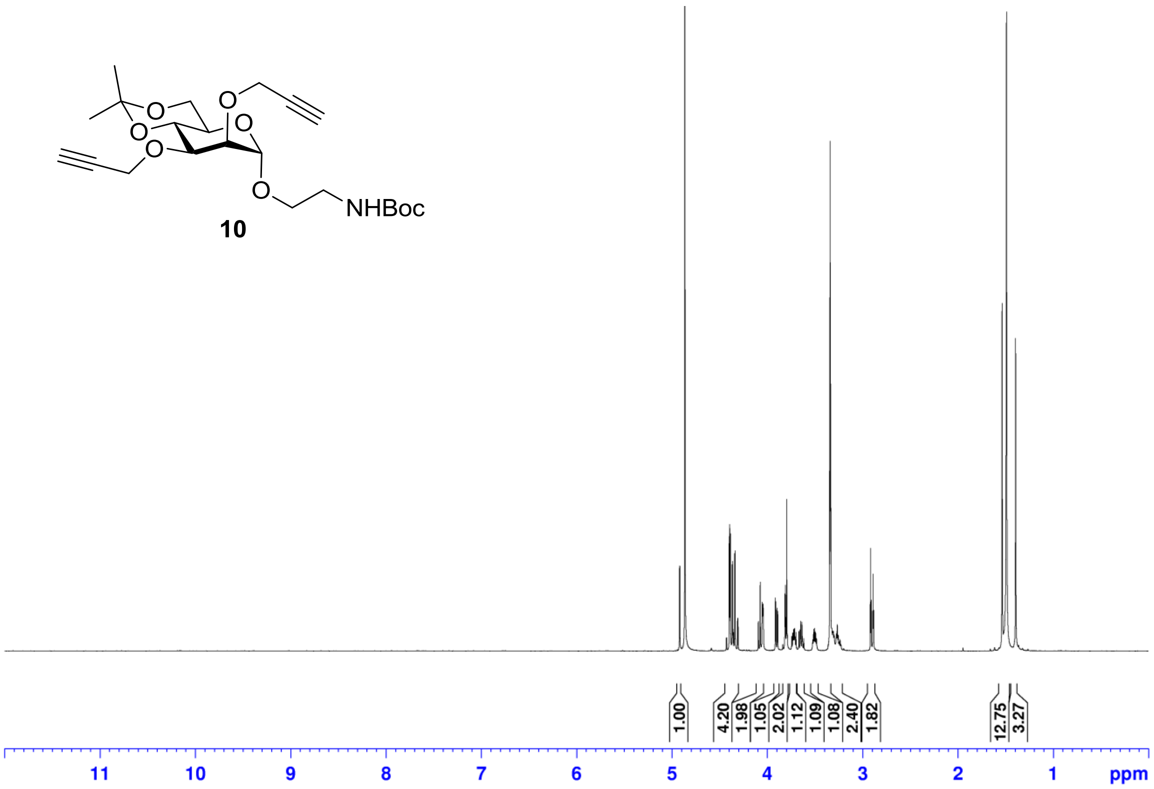

Figure S12: ^{1}H NMR spectrum (500 MHz, MeOH-d_4).

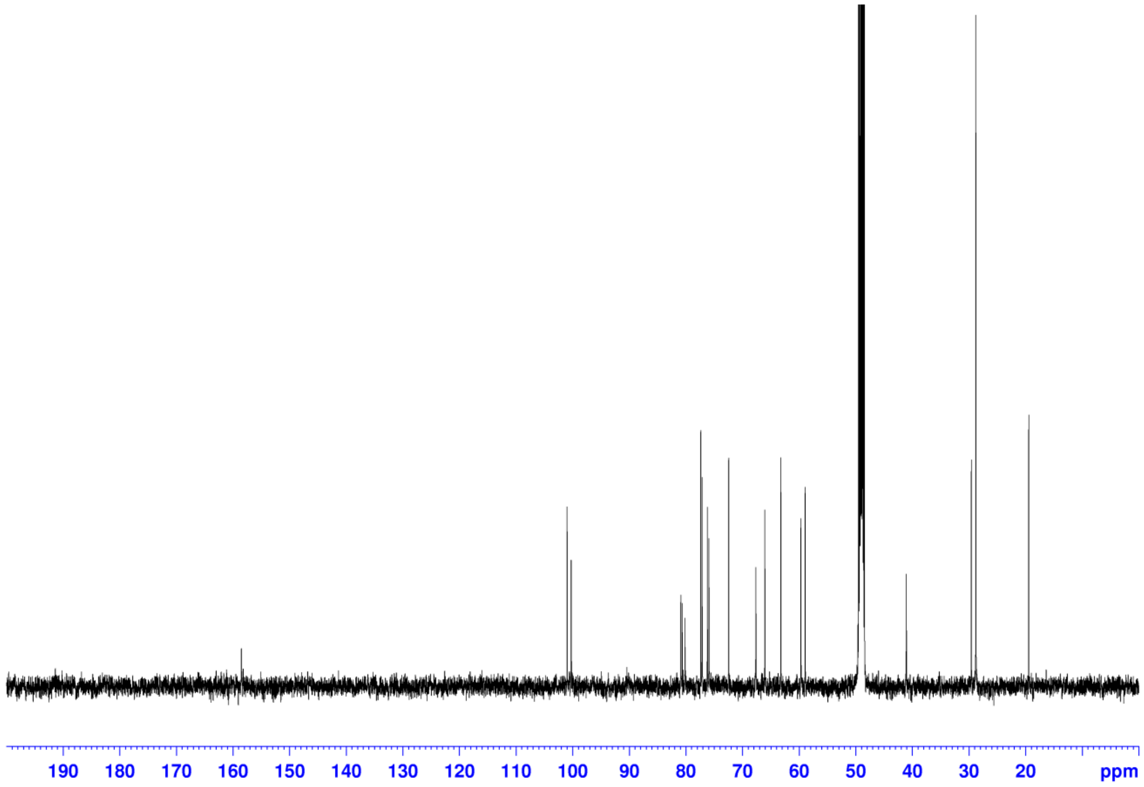

Figure S13: ^{13}C NMR spectrum (125 MHz, MeOH-d_4).

NMR spectra of **10**

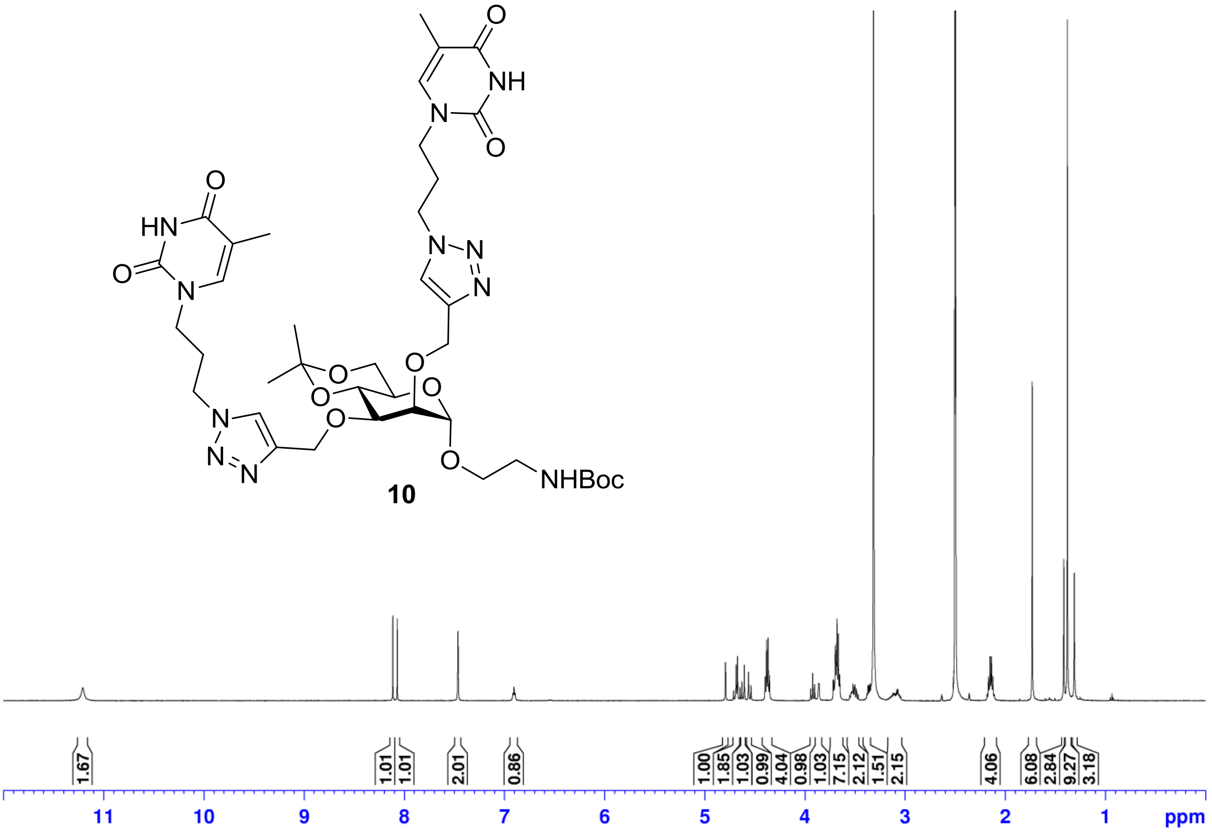

Figure S14: ^{1}H NMR spectrum (500 MHz, DMSO-d_6).

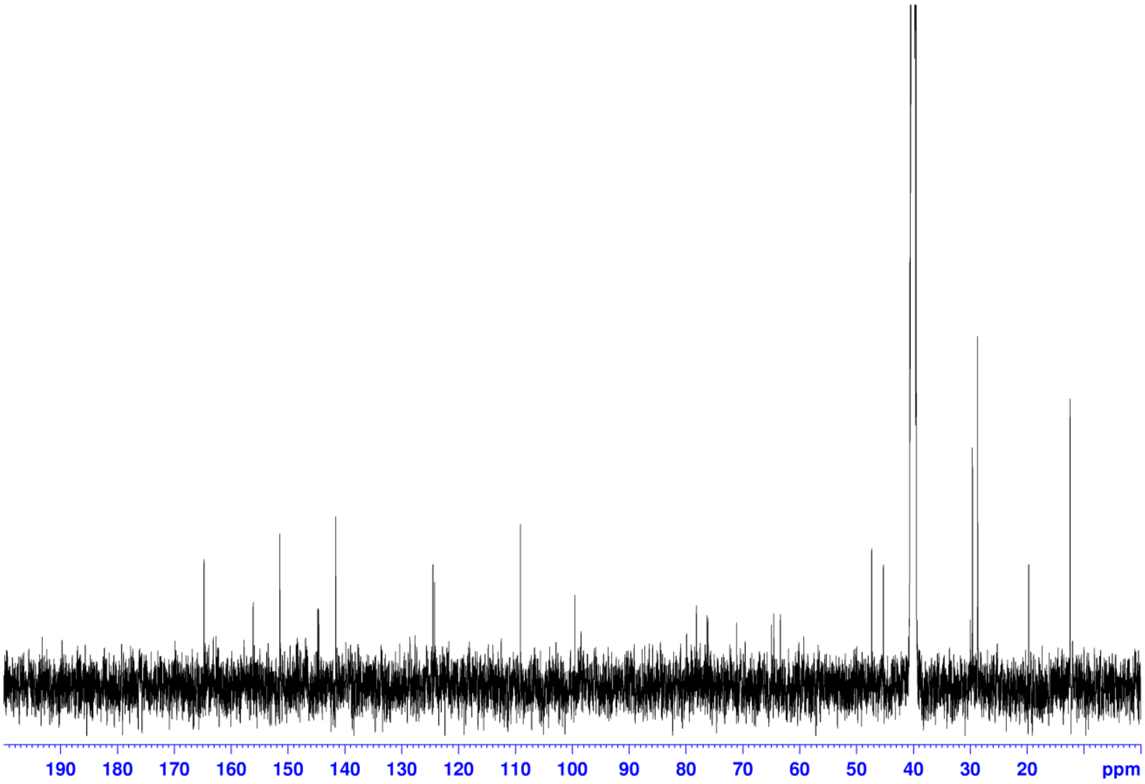

Figure S15: ^{13}C NMR spectrum (125 MHz, DMSO-d_6).

NMR spectra of **12**

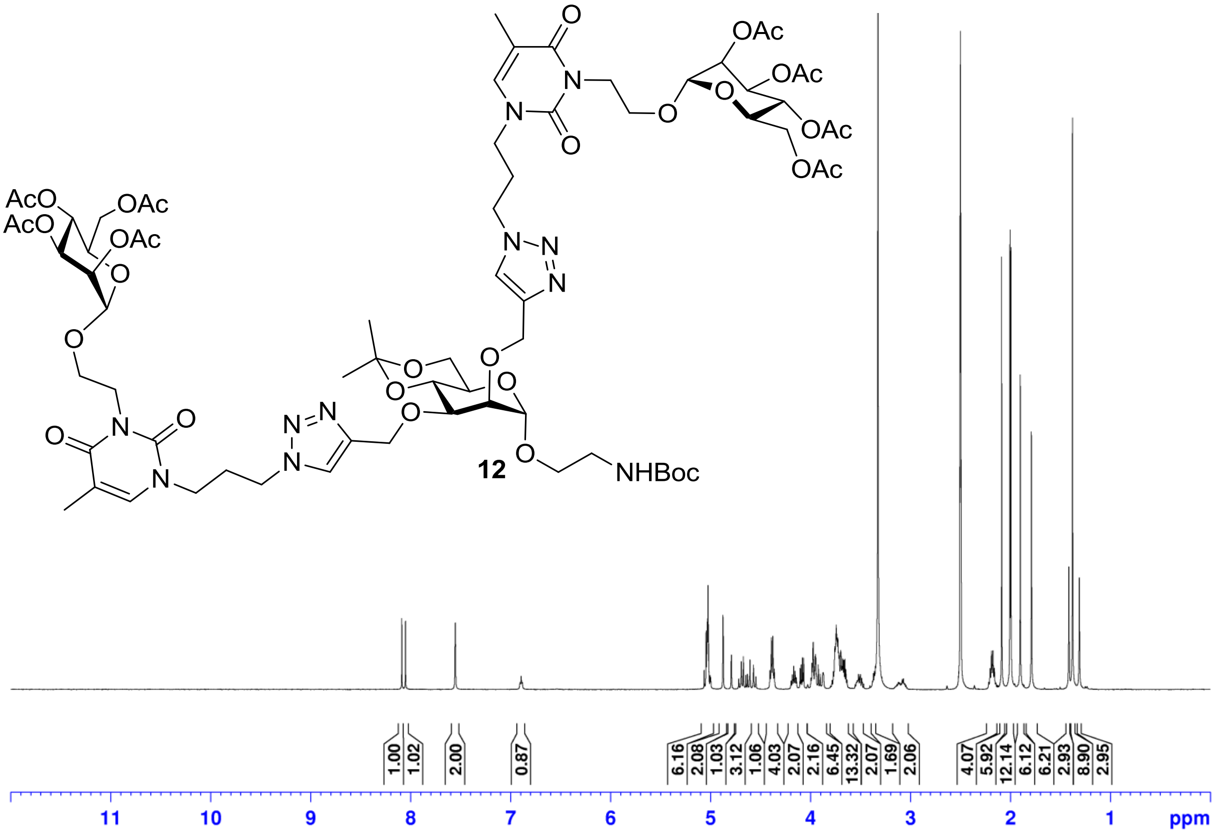

Figure S16: ^{1}H NMR spectrum (500 MHz, DMSO-d_6).

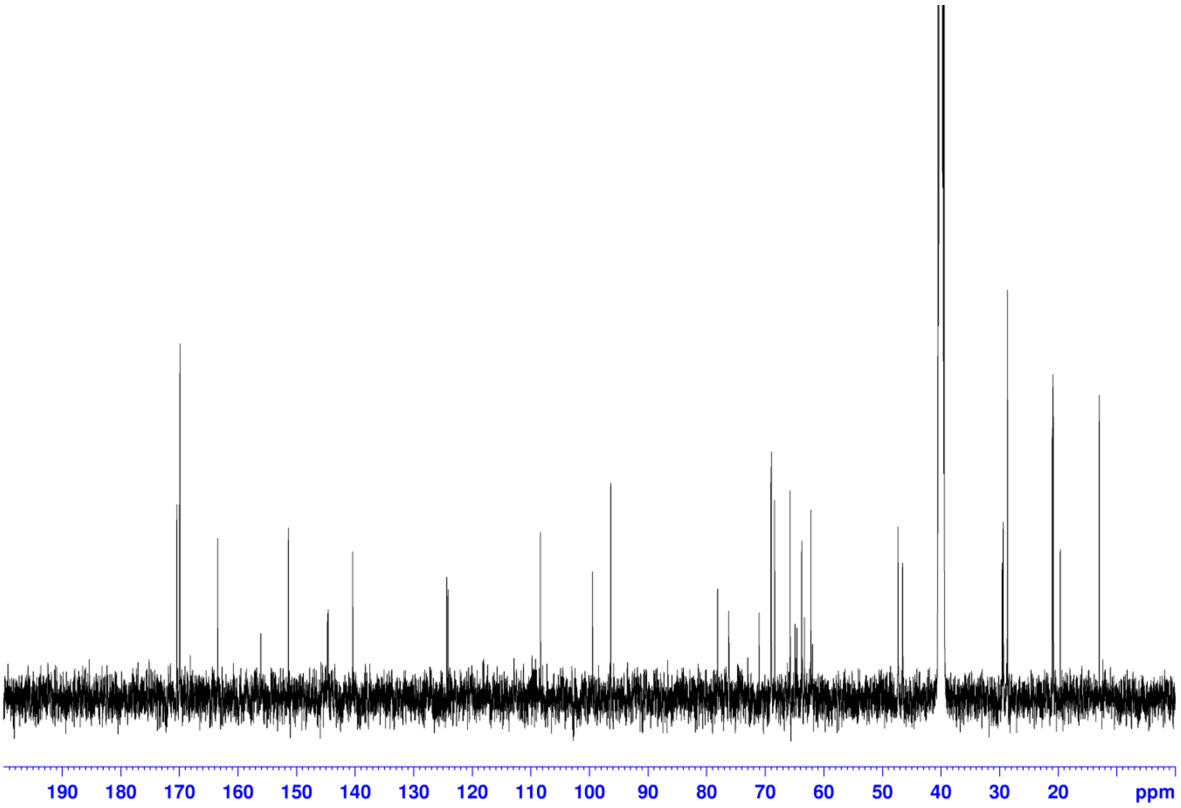

Figure S17: ^{13}CNMR spectrum (125 MHz, DMSO-d_6).

NMR spectra of **13**

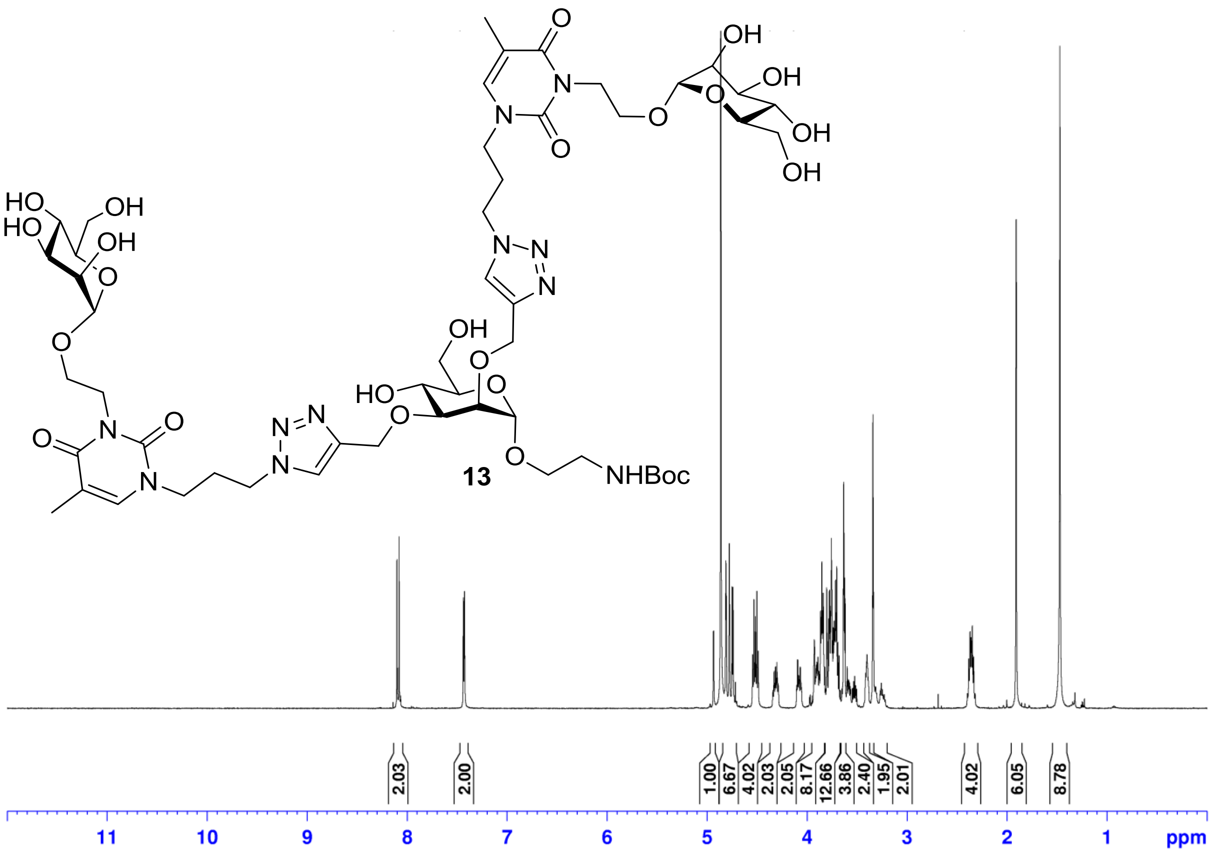

Figure S18: ^{1}H NMR spectrum (500 MHz, MeOH-d_4).

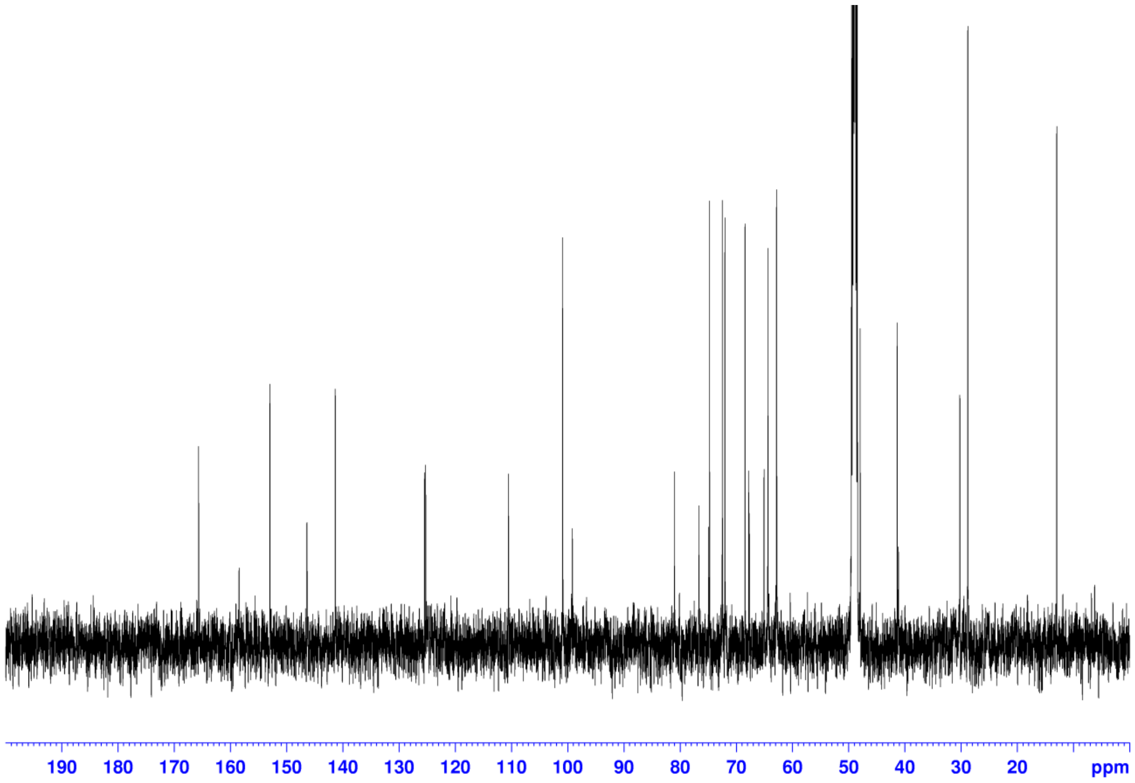

Figure S19: ^{13}C NMR spectrum (125 MHz, MeOH-d_4).

NMR spectra of **14**

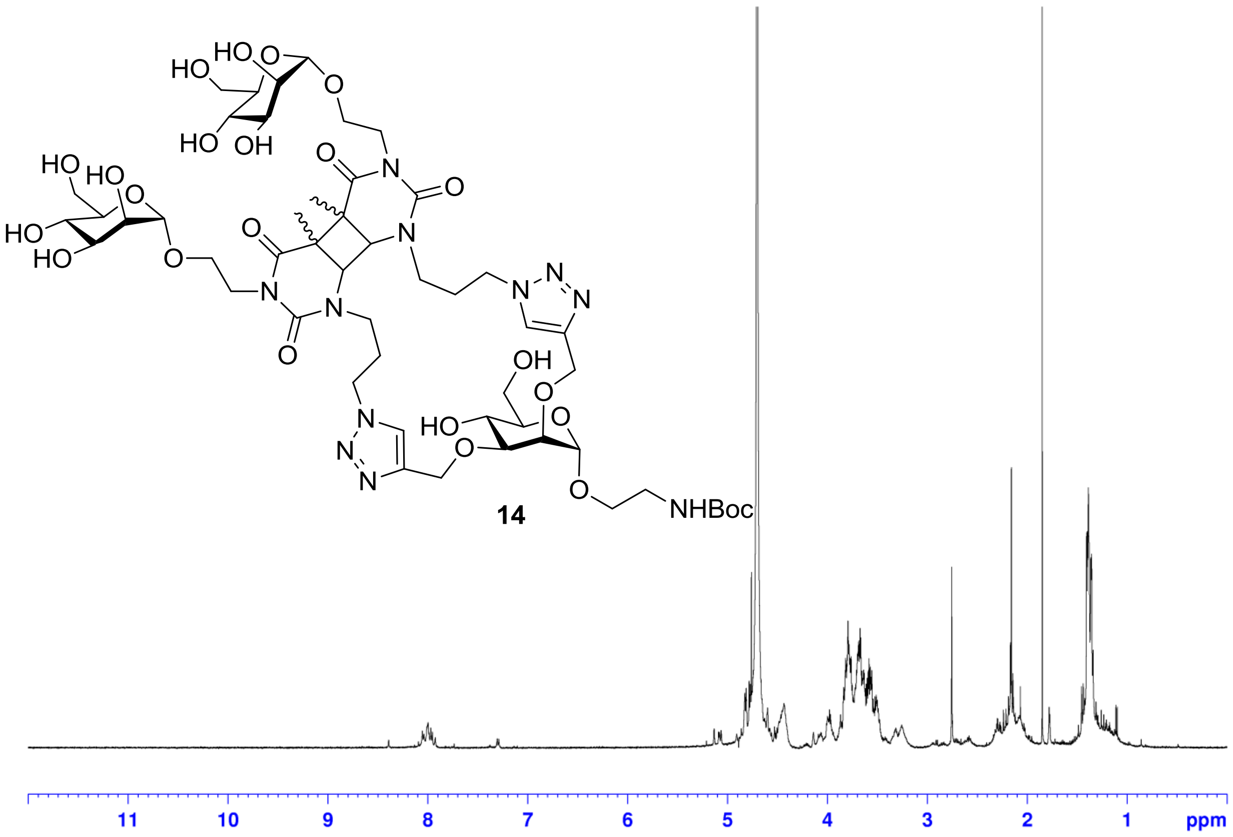

Figure S20: ^{1}H NMR spectrum of crude **14** (isomeric mixture) (500 MHz, D_2O).

Structures of all possible isomeric [2 + 2] photocycloaddition products resulting from intramolecular reaction (exemplified for irradiation of **7** leading to **8**).

The carbohydrate-scaffolded thymine derivatives can dimerize in *syn* or *anti* fashion leading to regioisomeric [2 + 2] photocycloaddition products. Each of the two regioisomeric products can form four different stereoisomers according to the relative steric position of the thymine methyl groups: two *cis* and two *trans* stereoisomers. The *syn*/*anti* – *cis*/*trans* nomenclature is used according to the literature (cf. Friedel, M. G.; Gierlich, J.; Carell, T. Cyclobutane Pyrimidine Dimers as Uv-Induced DNA Lesions. In *The chemistry of cyclobutanes*; Rappoport, Z.; Liebman, J. F., Eds.; Wiley and Sons: Chichester, U.K. 2005; pp 1031–1059).

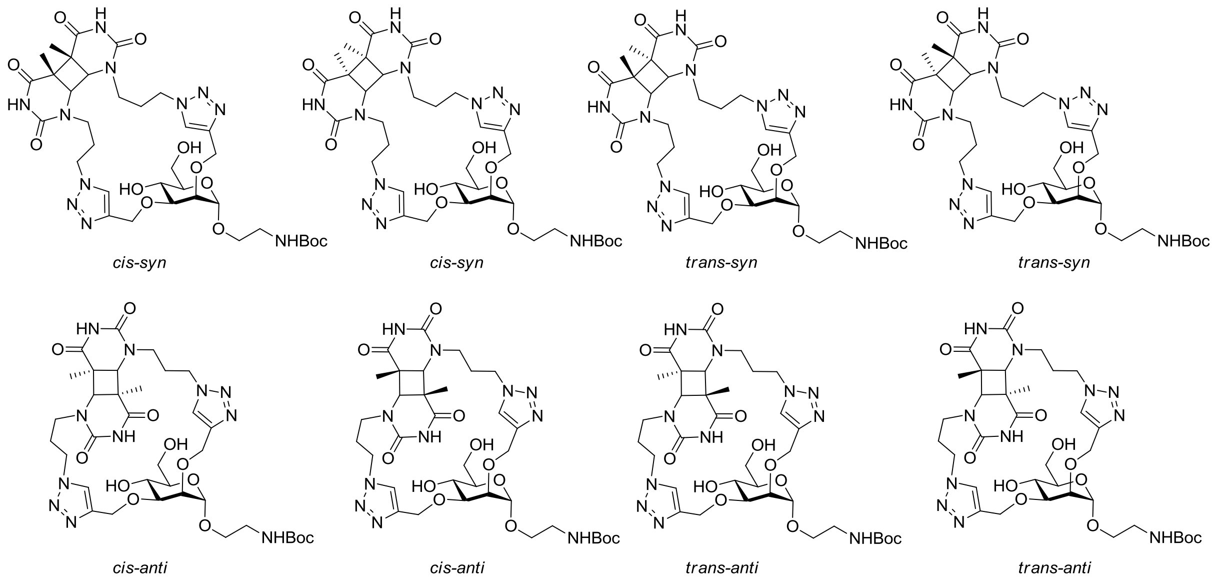

Figure S21: Structures of all possible [2 + 2] photocycloaddition isomers (**8**) resulting after irradiation of **7**.

UV–Vis spectroscopy of the [2 + 2] photocycloaddition of **7** leading to **8**.

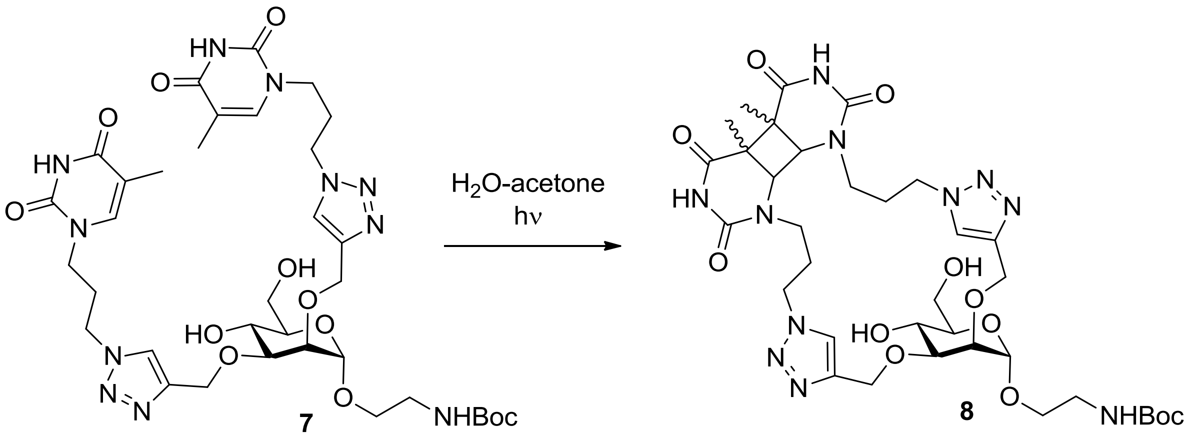

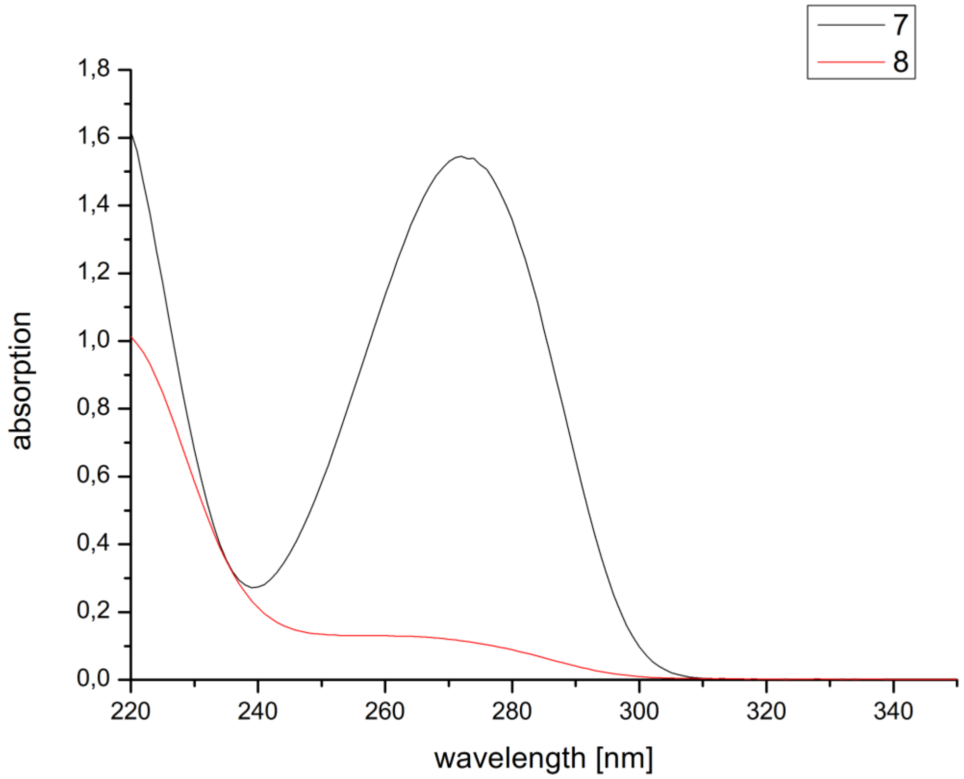

Figure S22: UV–vis spectra of divalent thymine conjugate **7** (conc. 100 µg/1 mL) and its [2 + 2] photocycloaddition product **8** (isomeric mixture) (conc. 100 µg/1 mL).

Mass spectra of **7** and **8**

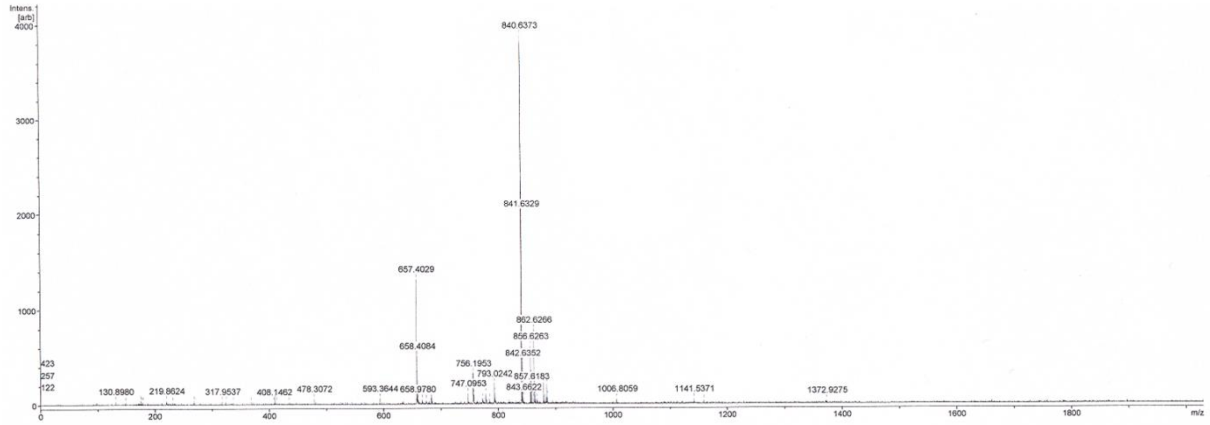

Figure S23: MALDI–ToF MS of **7**: $[M+Na]^+$ calcd. for $C_{35}H_{51}N_{11}O_{12}$: 840.3616; found *m/z* 840.6373 $[M+Na]^+$.

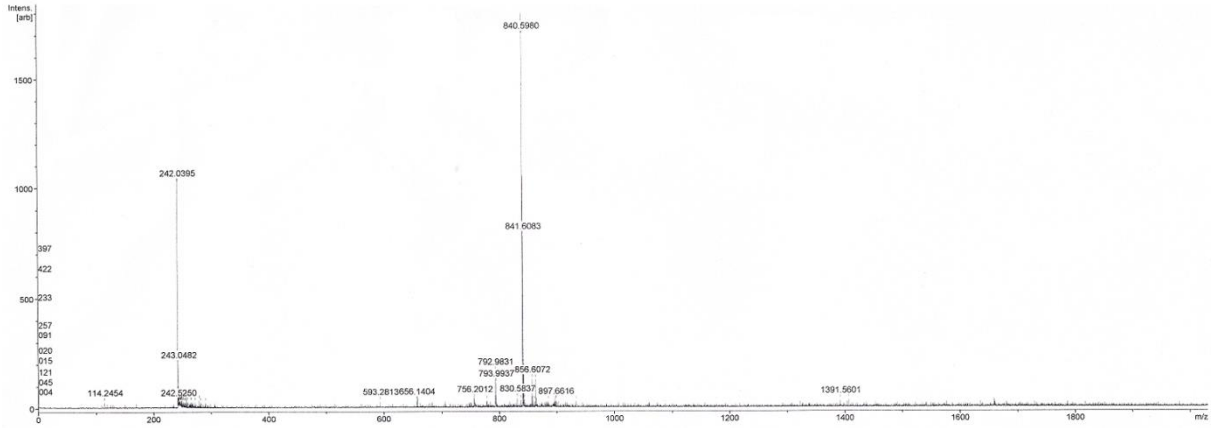

Figure S24: MALDI–ToF MS of **8** (isomeric mixture): $[M+Na]^+$ calcd. for $C_{35}H_{51}N_{11}O_{12}$: 840.3616; found *m/z* 840.5980 $[M+Na]^+$.

UV–vis spectroscopy of the [2 + 2] photocycloaddition of **13** leading to **14**.

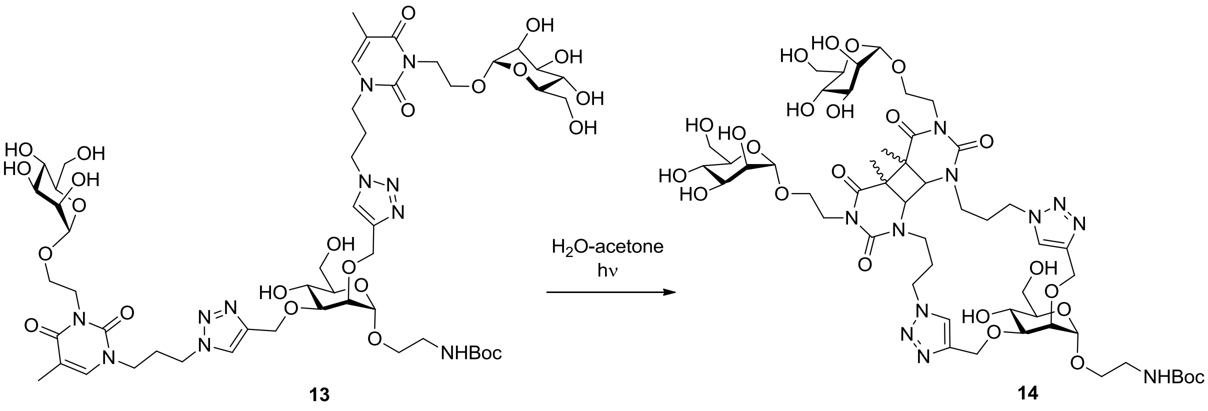

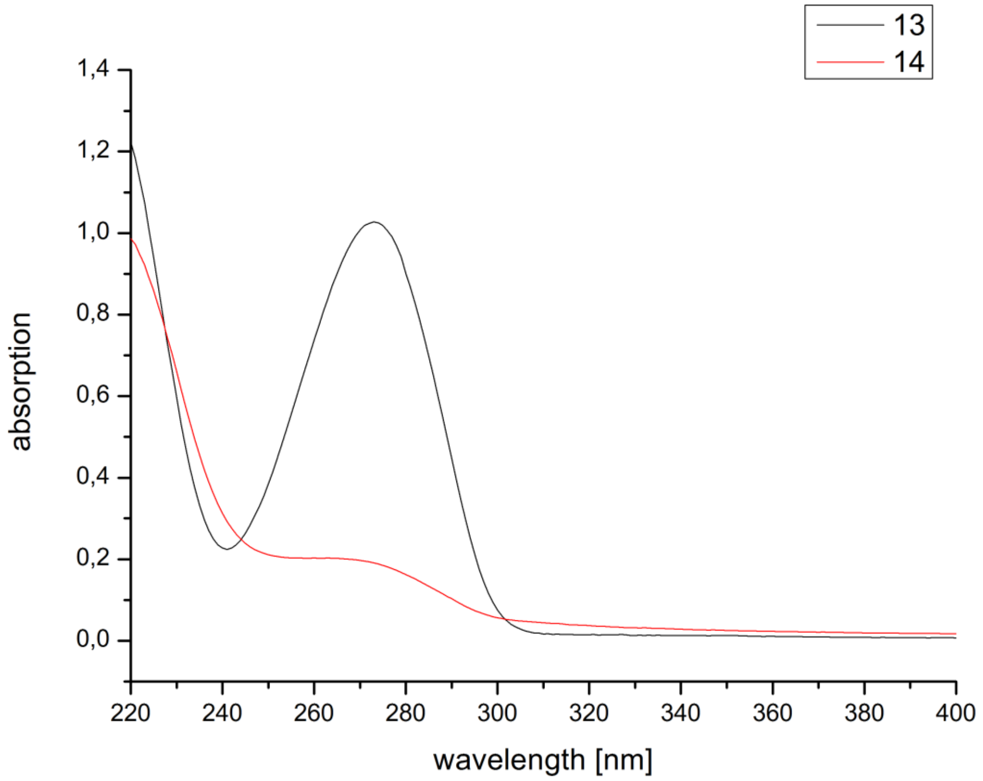

Figure S25: UV–vis spectra of divalent glycothymine conjugate **13** (conc. 100 µg/1 mL) and its [2 + 2] photocycloaddition product **14** (isomeric mixture) (conc. 100 µg/1 mL).

Mass spectra of **13** and **14**

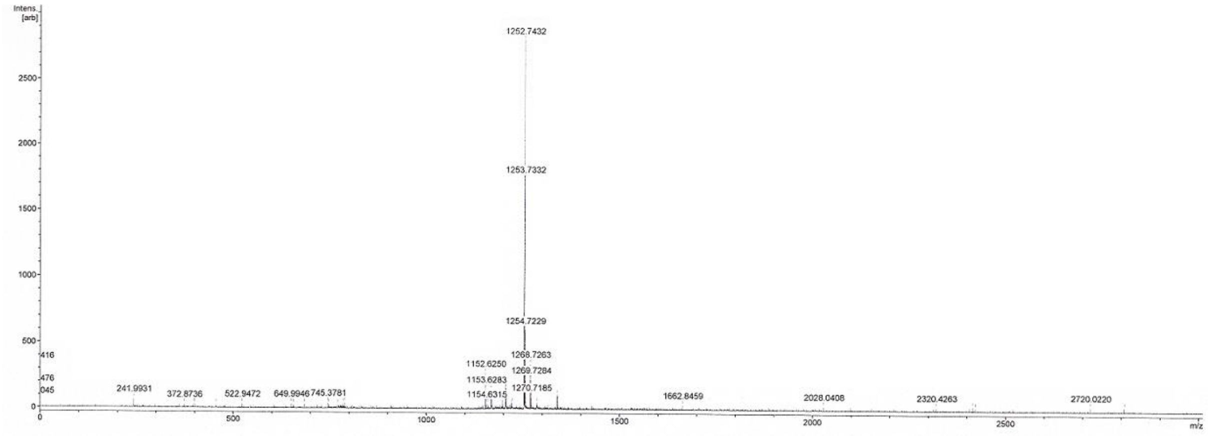

Figure S26: MALDI–ToF MS of **13**: $[M+Na]^+$ calcd. for $C_{51}H_{79}N_{11}O_{24}$: 1252.5197; found *m/z* 1252.7432 $[M+Na]^+$.

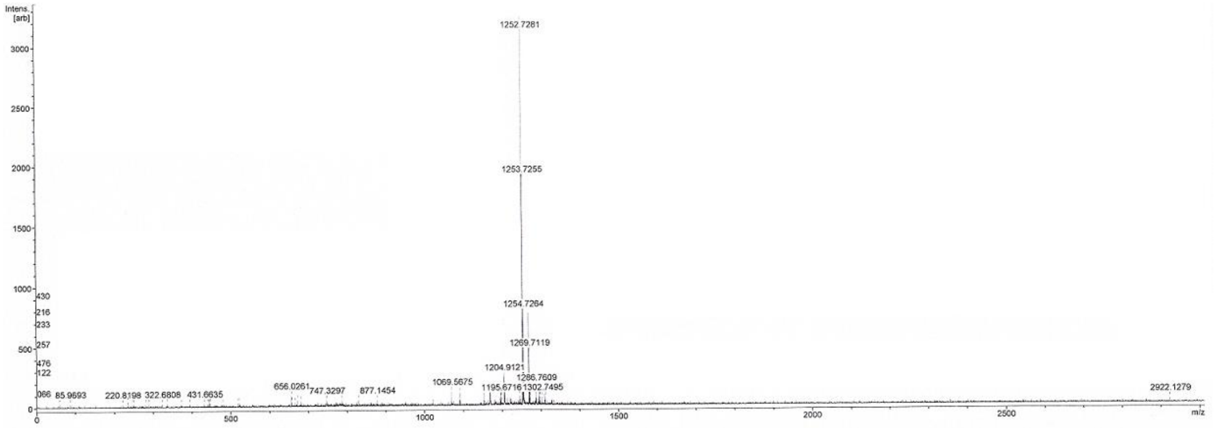

Figure S27: MALDI–ToF MS of **14** (isomeric mixture): $[M+Na]^+$ calcd. for $C_{51}H_{79}N_{11}O_{24}$: 1252.5197; found *m/z* 1252.7281 $[M+Na]^+$.
